# Identification of the Rage-dependent gene regulatory network in a mouse model of skin inflammation

**DOI:** 10.1186/1471-2164-11-537

**Published:** 2010-10-05

**Authors:** Astrid Riehl, Tobias Bauer, Benedikt Brors, Hauke Busch, Regina Mark, Julia Németh, Christoffer Gebhardt, Angelika Bierhaus, Peter Nawroth, Roland Eils, Rainer König, Peter Angel, Jochen Hess

**Affiliations:** 1 Signal Transduction and Growth Control, German Cancer Research Center (DKFZ), DKFZ-ZMBH Alliance, Heidelberg, Germany; 2 Theoretical Bioinformatics, German Cancer Research Center, Heidelberg, Germany; 3 Freiburg Institute for Advanced Studies - FRIAS School of Life Sciences - LIFENET Albert-Ludwigs-University Freiburg, Germany; 4 Center for Biosystems Analysis, Albert-Ludwigs-University Freiburg, Germany; 5 Institute of Pharmacy and Molecular Biology and Bioquant Center, University of Heidelberg, Germany; 6 Department of Dermatology, University Hospital Heidelberg, Germany; 7 Department of Medicine I and Clinical Chemistry, University Hospital Heidelberg, Germany; 8 Experimental Head and Neck Oncology, Department of Otolaryngology, Head and Neck Surgery, University Hospital Heidelberg, Germany

## Abstract

**Background:**

In the past, molecular mechanisms that drive the initiation of an inflammatory response have been studied intensively. However, corresponding mechanisms that sustain the expression of inflammatory response genes and hence contribute to the establishment of chronic disorders remain poorly understood. Recently, we provided genetic evidence that signaling via the receptor for advanced glycation end products (Rage) drives the strength and maintenance of an inflammatory reaction. In order to decipher the mode of Rage function on gene transcription levels during inflammation, we applied global gene expression profiling on time-resolved samples of mouse back skin, which had been treated with the phorbol ester TPA, a potent inducer of skin inflammation.

**Results:**

Ranking of TPA-regulated genes according to their time average mean and peak expression and superimposition of data sets from wild-type (*wt*) and *Rage*-deficient mice revealed that Rage signaling is not essential for initial changes in TPA-induced transcription, but absolutely required for sustained alterations in transcript levels. Next, we used a data set of differentially expressed genes between TPA-treated *wt *and *Rage*-deficient skin and performed computational analysis of their proximal promoter regions. We found a highly significant enrichment for several transcription factor binding sites (TFBS) leading to the prediction that corresponding transcription factors, such as Sp1, Tcfap2, E2f, Myc and Egr, are regulated by Rage signaling. Accordingly, we could confirm aberrant expression and regulation of members of the E2f protein family in epidermal keratinocytes of Rage-deficient mice.

**Conclusions:**

In summary, our data support the model that engagement of Rage converts a transient cellular stimulation into sustained cellular dysfunction and highlight a novel role of the Rb-E2f pathway in Rage-dependent inflammation during pathological conditions.

## Background

A striking feature of many human cancers is an underlying and unresolved inflammation, which often predates the disease and orchestrates a tumor supporting microenvironment. Indeed, several lines of evidence, including population-based epidemiological and clinical studies as well as experimental animal model systems, highlighted chronic infection and persistent inflammation as major risk factors for various types of cancer [1,2]. Thus, molecular mechanisms converting a transient inflammatory tissue reaction into a tumor promoting microenvironment as well as signaling and gene regulatory networks implicated in cellular communication between tumor and immune cells will be auspicious targets for innovative strategies of translational cancer research.

Recently, we could show that the receptor for advanced glycation end products (Rage) drives the strength and maintenance of inflammation during tumor promotion in a mouse model of inflammation-associated skin carcinogenesis [3]. Accordingly, tumor formation in mutant mice with *Rage *deletion (*Rage^-/-^*) was impaired in this model, but also in a tumor model of colitis-induced colon cancer [3,4].

Rage is a multi-ligand as well as pattern recognition receptor of the immunoglobulin super-family with low expression levels in most adult tissues. However, Rage expression increases at sites of inflammation, mainly on inflammatory cells, endothelial cells and epithelial cells, and propagates cellular dysfunction in numerous inflammation-related pathological states, such as diabetes, vascular disease, neurodegeneration, chronic inflammation, and cancer [5-7].

With respect to Rage signaling, several target genes have been identified in the past, including pro-inflammatory mediators, matrix metalloproteinases, and adhesion proteins, however, their expression critically depends on the cell type, its microenvironment, and quality of the stimulus [8]. In the process of neoplastic transformation and malignant progression, activation of Rage by its ligands, such as advanced glycation end products (AGEs), high mobility group box-1 (Hmgb1), and members of the S100 protein family, can stimulate tumor cell proliferation, invasion, chemoresistance, and metastasis [9-11]. Rage ligands derived from cancer cells can also support the establishment of a pro-tumorigenic microenvironment by activation of leukocytes, vascular cells, fibroblasts, and modulation of immune tolerance [11]. Although multiple intracellular signaling pathways, including MAP kinases, Rho GTPases, PI3K, JAK/STAT, and NF-κB, have been found to be altered following Rage stimulation, the molecular mechanisms how Rage triggers intracellular signaling to regulate cellular decisions remain largely elusive, and the identity of direct signaling molecules downstream of the receptor are still unknown [5,12-14].

In order to elucidate how Rage receptor signaling converts a transient stimulus into a long lasting response, global gene expression kinetics were recorded with skin samples of *wt *and *Rage^-/- ^*mice upon TPA stimulation. We applied a recently published computational analysis tool that enables a global, holistic view on cellular responses over a time frame of hours based on dynamic transcription level data [15], and identified the characteristic duration and temporal order of transient and Rage-dependent events upon TPA stimulation. Subsequently, a computational approach was applied to predict transcription factors that are implicated in the Rage-dependent regulation of pro-inflammatory gene expression, and thus, to identify novel key molecules as putative targets for innovative strategies of anti-inflammatory therapy.

## Results

### Identification of Rage-dependent gene expression upon TPA treatment of mouse back skin

**Figure 1:**
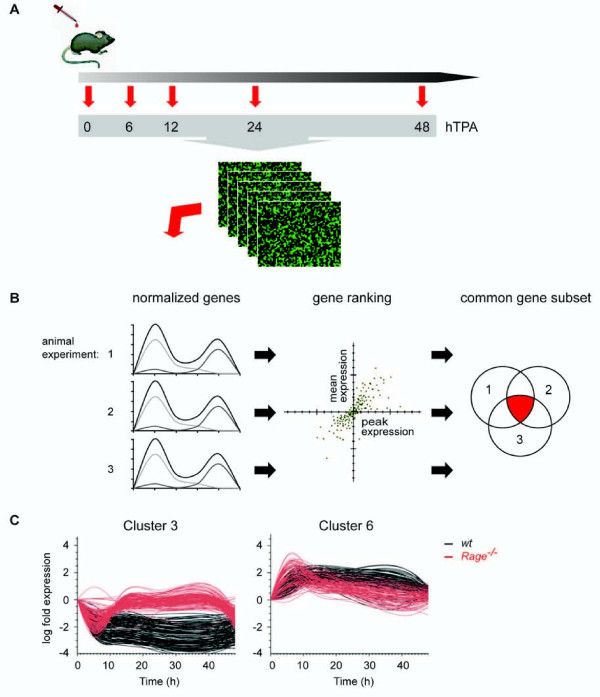
**Global gene expression analysis of *wt *and *Rage^-/- ^*skin following single TPA treatment**. (A) Mice of respective phenotypes were treated once with TPA, and back skin was isolated 6, 12, 24, or 48 hours after stimulation. Non-treated and acetone-treated mice served as control (0). Global gene expression analysis of RNA samples was performed on whole mouse genome oligonucleotide microarrays (n = 3 for each genotype and time point). (B) Following quantile normalization, *wt *genes of each kinetic from three independent animal experiments (1, 2, and 3) were ranked according to high mean and peak expression separately to filter for TPA-responsive genes. A common subset of 341 genes was identified out of the top 1000 ranked genes within each kinetic. (C) K-means clustering revealed 6 clusters of which cluster 3 and 6 shared most genes (for cluster 1, 2, 4, and 5 see Additional file 2). Black lines represent transcript levels of TPA-regulated genes in *wt *samples and red lines represent the corresponding genes in *Rage^-/- ^*samples.

In order to identify alterations in the gene expression profile during the process of skin inflammation we applied TPA on the back skin of *wt *and *Rage^-/- ^*mice and prepared total RNA at consecutive time points after treatment (6, 12, 24, and 48 hours following TPA application in three individual animal experiments). The RNA was hybridized on whole mouse genome oligonucleotide microarrays followed by feature extraction and quantile normalization procedure (Figure 1A). The gene fold expression was calculated with respect to non-treated controls (0 h), and TPA-responsive genes in samples of *wt *back skin were ranked according to their combined averaged mean and peak expression within the experimental time window of 48 hours for each individual kinetic series. Subsequently, we identified a common subset of 341 genes among the 1,000 highest ranked genes in all three experiments with a small variance between the experiments (Figure 1B and see Additional file 1). These genes were further separated into six expression profile sets according to k-means clustering (see Additional file 2). Most candidate genes were found in cluster 3 (n = 125) or in cluster 6 (n = 84), representing genes that were either TPA-repressed or TPA-induced within 6 hours and maintained altered expression for at least 24 hours (Figure 1C). Interestingly, when we considered the transcript levels of these genes in *Rage^-/- ^*back skin and superimposed both *wt *and *Rage^-/- ^*data sets we found a comparable response in both genotypes at 6 hours. However, initial transcript level responses ceased to basal levels in *Rage^-/- ^*skin between 12 and 24 hours upon stimulation, whereas the response was sustained in *wt *animals. Our data suggest the existence of two phases of the TPA response: an initial Rage-independent response that is followed by a second Rage-dependent maintenance of the altered transcript levels (Figure 1C).

Next, linear models with empirical Bayesian correction were applied to identify differentially expressed genes between *wt *and *Rage^-/- ^*back skin at the investigated time points after TPA administration. In line with preceding analyses and previous results [3], genes (n = 122) that differ significantly between both genotypes were only found 24 hours after TPA stimulation (see Additional file 3). According to their temporal expression pattern, differentially expressed genes were further divided by unsupervised hierarchical clustering of their correlation distance (one minus the Pearson correlation coefficient) into three sub-clusters. While cluster 1 (n = 52) and cluster 2 (n = 25) shared TPA-induced genes, cluster 3 (n = 45) was composed of TPA-repressed genes (see Additional file 3). We selected several differentially expressed genes (*Tgfb1, Tnf, Fosl1, Mmp2, Irf7, Hmgb2*, and *Hdac2*) and could confirm altered transcript levels by quantitative real-time PCR using cDNA from back skin of *wt *and *Rage^-/- ^*mice 24 hours after TPA treatment (see Additional file 4). With regard to their functional annotation, genes of cluster 1 were correlated with immune effector process, tissue remodeling and cell signaling, while genes of cluster 3 showed evident connection to histone and chromatin modifications as well as metabolic processes (data not shown).

Taken together, time-resolved global gene expression analysis of *wt *and *Rage^-/- ^*skin upon TPA application disclosed expression patterns that subdivide the TPA-induced response into an initial Rage-independent phase and a second Rage-dependent maintenance of the established signal. Differentially expressed genes 24 hours after TPA stimulation revealed three gene clusters characterized by distinct functions.

### Prediction of transcription factors implicated in the Rage-dependent gene regulatory network

**Figure 2:**
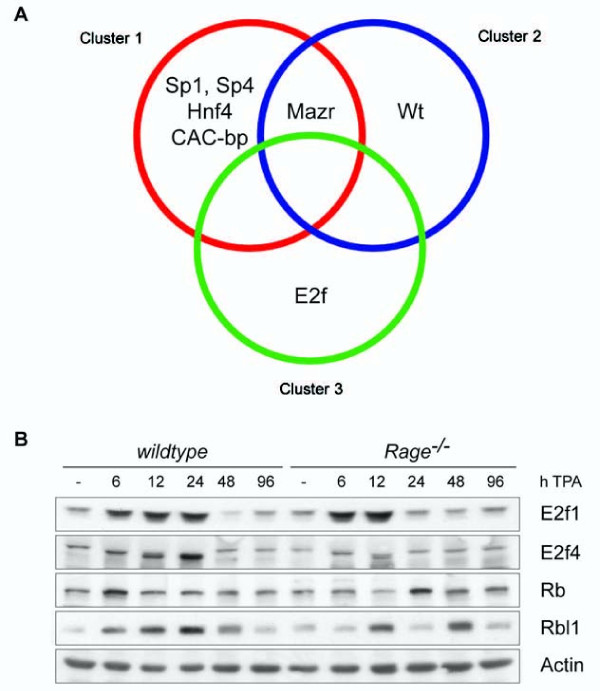
***In silico *promoter analysis of differentially expressed genes 24 hours after TPA treatment**. (A) Annotated promoter sequences of 97 differentially expressed genes between *wt *and *Rage^-/- ^*skin were screened for significant enrichment of transcription factor binding sites (TFBS). TFBS were grouped according to their enrichment within clusters of differentially expressed genes between *wt *and *Rage^-/- ^*skin 24 hours after TPA treatment. (B) Protein expression of E2f1, E2f4, Rb, and Rbl1 was investigated by Western blot analysis with whole cell lysates of TPA- and control-treated back skin of *wt *and *Rage^-/- ^*mice. Actin protein levels served as control for protein quality and quantity.

In order to identify relevant transcription factors implicated in the regulation of Rage-dependent genes we performed an *in silico *promoter analysis. We used the probes that were differentially expressed between *wt *and *Rage^-/- ^*mice at the time point 24 hours after TPA application and selected those that mapped unambiguously to one Entrezgene-ID and for which the promoter sequence was available (n = 97). These probes were clustered by their correlation distance within the samples from t = 24 hours into three clusters (see Additional file 5). We analyzed 2 kb upstream and downstream sequences of the annotated transcriptional start site and calculated the enrichment of transcription factor binding sites (TFBS) compared to all other available genes represented on the microarray by Fisher's exact tests. The analysis revealed several highly enriched TFBS for Specificity protein 1 and 4 (Sp1 and 4), Activator protein 2 (Ap2/Tcfap2), E2-promoter-binding factor (E2f), Myc-associated zinc-finger protein and Myc-associated zinc-finger protein-related protein (Mazr), Early growth response factor (Egr), CAC-binding protein (CAC-bp), v-Myc myelocytomatosis viral oncogene homolog (Myc), Nuclear receptor subfamily 2 group F members (Nr2f/COUP-TF), and Wilms tumor 1 homolog (Wt1) (Table 1). The enrichment tests were also applied on each of the three clusters separately to address the question, whether specific TFBS were significantly associated with differentially expressed genes in distinct clusters. While TFBS for Mazr were enriched in promoters of genes of at least 2 of 3 clusters, a significant correlation of TFBS for Sp1, Sp4, Hnf4, and CAC-bp were only found for promoters of genes in cluster 1 (Figure 2A). Similarly, significant enrichment of TFBS for Wt1 was restricted for gene promoters in cluster 2, and TFBS for E2f were limited to gene promoters in cluster 3 (Figure 2A).

**Table 1:** *In silico *promoter analysis of differentially expressed genes 24 hours after TPA stimulation

Genes	BF Name	Fischertest P.Val	Corrected P.Val	With PWM cluster	Without PWM cluster		
all	Sp1	5.33E-07	1.06E-04	94	3		
	Sp1 isoform 1	5.33E-07	1.06E-04	94	3		
	Sp4	1.72E-06	2.27E-04	83	14		
	AP-2beta	1.24E-06	1.22E-03	77	20		
	AP-2alpha	1.60E-05	1.27E-03	79	18		
	AP-2gamma	2.13E-05	1.41E-03	79	18		
	MAZR	6.03E-05	3.41E-03	74	23		
	CAC-binding protein	1.32E-04	6.52E-03	81	16		
	Egr-1	3.56E-04	1.56E-02	85	12		
	Egr-3	4.38E-04	1.73E-02	78	19		
	E2F	4.85E-04	1.75E-02	58	39		
	c-Myc	7.31E-04	2.41E-02	67	30		
	Egr-2	9.69E-04	2.94E-02	80	17		
	COUP-TF1	1.06E-03	2.94E-02	89	8		
	WT1	1.19E-03	2.94E-02	67	30		
	WT1-isoform1	1.19E-03	2.94E-02	67	30		
	COUP-TF2	1.45E-03	3.38E-02	48	49		
Cluster 1	Sp4	2.01E-06	7.93E-04	40	2		
	Sp1	6.13E-05	6.86E-03	42	0		
	Sp1 isoform 1	6.13E-05	6.86E-03	42	0		
	MAZR	6.93E-05	6.86E-03	36	6		
	HNF-4alpha7	1.84E-04	1,46E-02	29	13		
	CAC-binding protein	3.12E-04	2.06E-02	38	4		
Cluster 2	MAZR	2.14E-04	7.64E-02	19	1		
	WT1	5.79E-04	7.64E-02	18	2		
	WT1-isoform1	5.79E-04	7.64E-02	18	2		
Cluster 3	E2F	1.88E-05	7.45E-03	28	8		
	E2F-1	8.50E-05	1.68E-02	28	8		

In summary, the enrichment analyses highlighted the putative involvement of several transcription factors, such as E2f and Wt1, that were previously not associated with Rage signaling, and therefore, represent an exciting starting point for further investigation.

### Impact of Rage on the regulation of E2f proteins in TPA-treated skin

To confirm our prediction on transcription factors implicated in Rage-dependent gene transcription, we investigated the expression and regulation of the E2f transcription factor that is well known to determine cellular responses to growth factors, stress and differentiation signals, as well as DNA damage [16]. E2f represents a family of eight transcription factors that are further subdivided into a group of potent transcriptional activators (E2f1-3a) and a group of preferential transcriptional repressors (E2f3b, E2f4-8) [17]. Analysis of our global gene expression data revealed no major alteration in transcript levels of most E2f family members between both genotypes, suggesting an impact of Rage on posttranslational regulation of E2f proteins. Interestingly, we found strong induction in protein levels of E2f1, a representative for the group of transcriptional activators, 6 and 12 hours after TPA application in skin lysates of both genotypes. However, enhanced protein levels 24 hours after TPA stimulation were only detected in *wt *lysates (Figure 2B). E2f4, a representative for the group of transcriptional repressors, gradually increased upon TPA treatment, showing a peak at 24 hours in *wt *skin, while no alterations in protein level were detected in *Rage^-/- ^*samples throughout the kinetic (Figure 2B). Retinoblastoma (Rb) and retinoblastoma-like (Rbl) proteins regulate the activity of E2f transcription factors [16]. We found changes for Rb protein expression with highest levels 6 hours after stimulation in *wt *skin samples and 24 hours in *Rage^-/- ^*skin samples (Figure 2B). Rbl1 protein expression was induced in *wt *and *Rage^-/- ^*skin samples following TPA stimulation, but a concerted increase over time was only detected for *wt *animals. Together, these data support the conception that the Rb-E2f pathway is downstream of Rage signaling and critically contributes to altered gene transcription during TPA-induced skin inflammation.

**Figure 3:**
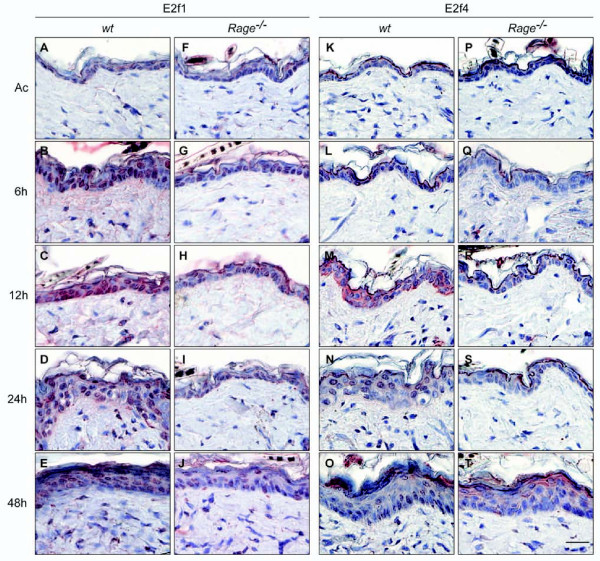
**E2f1 and E2f4 protein expression in skin following a single TPA stimulus**. Tissue sections of acetone- (Ac) or TPA-treated (6, 12, 24, and 48 hours) back skin from *wt *and *Rage^-/- ^*mice were analyzed by immunohistochemical staining using E2f1- and E2f4-specific antibodies. Representative images of at least 2 animals of each genotype and time point are shown with red staining for E2f1 or E2f4 and counterstaining with hematoxylin. Scale bar = 25 μm.

**Figure 4:**
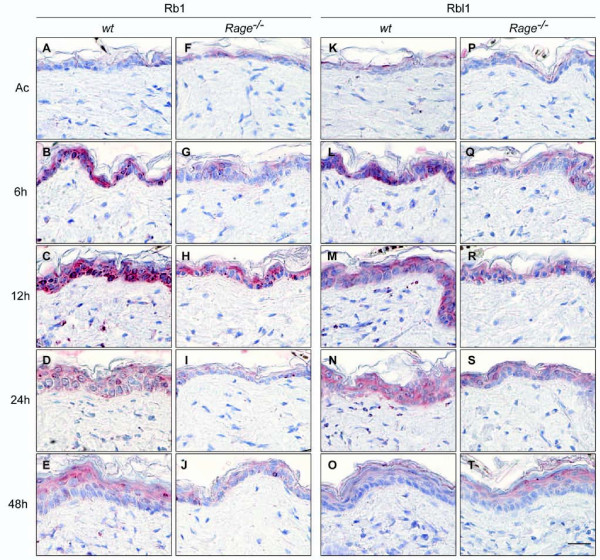
**Rb and Rbl1 protein expression in skin following a single TPA stimulus**. Tissue sections of acetone- (Ac) or TPA-treated (6, 12, 24, and 48 hours) back skin from *wt *and *Rage^-/- ^*mice were analyzed by immunohistochemical staining using Rb- and Rbl1-specific antibodies. Representative images of at least 2 animals of each genotype and time point are shown with red staining for Rb or Rbl1 and counterstaining with hematoxylin. Scale bar = 25 μm.

Immunohistochemical staining was performed on tissue sections of *wt *and *Rage^-/- ^*back skin upon single TPA treatment in order to investigate whether keratinocytes were the cellular origin of altered E2f protein levels. While slight staining for E2f1 protein was detected in keratinocytes of control-treated *wt *back skin, intense nuclear staining was found in kerationcytes upon TPA stimulation (Figure 3A-E). A similar staining pattern for E2f1 protein was observed in control-treated *Rage^-/- ^*back skin and 6 hours after TPA administration, however, less intense staining was determined at later time points (Figure 3F-J). Immunohistochemical analysis of E2f4 protein revealed a strong but transient induction in the cytoplasm of keratinocytes of *wt *back skin 12 hours after TPA stimulation, followed by translocation of E2f4 protein into the nucleus by 24 hours after TPA application (Figure 3K-O). Again, an obvious change in E2f4 protein expression was detectable in *Rage^-/- ^*back skin (Figure 3P-T). These data are in clear accordance with our immunoblot data and demonstrate a direct correlation between RAGE signaling and E2f-dependent gene expression in epidermal keratinocytes upon TPA-induced skin inflammation. Finally, we also determined Rb, Rbl1, and Rbl2 protein levels by immunochistochemistry. While no major alteration in Rbl2 protein levels was observed following TPA stimulation or between both genotypes (see Additional file 6), we found a stronger staining for Rb and Rbl1 proteins in keratinocytes of TPA-treated *wt *compared to *Rage^-/- ^*back skin (Figure 4).

## Discussion

The aim of our study was to highlight the molecular mechanism how Rage signaling contributes to the dynamic long-term gene regulatory response under physiological and pathological conditions of inflammation. We selected the model of TPA-induced inflammation on mouse back skin since it allows a highly reproducible and temporal analysis of altered gene expression during acute phase inflammation, including the initiation as well as the resolution phase. Furthermore, we recently provided experimental evidence that *Rage^-/- ^*mice are defective in the establishment and maintenance of dermal inflammation upon TPA stimulation accompanied by impaired tumor formation in a chemically induced skin tumor model [3]. Finally, it is worthwhile to note that Rage and its ligands are expressed or induced in numerous cell types, including keratinocytes, immune cells and endothelial cells [5]. With regard to this complex autocrine and paracrine signaling, *in vitro *approaches to identify the role of Rage signaling on gene regulatory networks under pathological conditions are almost impossible.

Global profiling of gene expression kinetics with samples from TPA-treated *wt *mice revealed a comprehensive list of differentially expressed genes that were strongly induced or repressed within 6 hours and maintained altered transcript levels for at least 24 hours. Intriguingly, most of these genes exhibited similar changes in expression at early time points, but rapidly returned to basal levels in the absence of Rage, providing experimental evidence that Rage is not necessarily required to initiate gene regulation in TPA-induced skin inflammation. However, Rage is absolutely required to maintain altered expression of genes implicated in immune effector processes, cell signaling, as well as histone and chromatin organization, and thereby sustains the tissue response.

Our data fit into the model that engagement of Rage converts a transient cellular stimulation into sustained cellular dysfunction. An important driver of this conversion is the long-term activation of pro-inflammatory transcription factors, especially NF-κB, which represents a key feature of most intracellular signaling pathways that have been described downstream of Rage stimulation [6,8,18]. However, we hypothesized that in addition to NF-κB other transcription factors contribute to Rage-dependent modulation of the gene regulatory network. Obviously, most genes with a significant difference in transcript levels between *wt *and *Rage^-/- ^*back skin were detectable 24 hours after TPA administration due to the effect of Rage on the temporal expression pattern. Computational analysis of TFBS in proximal promoter regions of differentially expressed genes allowed prediction of specific transcription factors that act downstream of Rage signaling during TPA-induced inflammation. In line with our data, a couple of recent studies describe a direct link between Rage signaling and Egr-1 activation in endothelial cells and liver cells [19-22]. In this context, Egr-1 was found in the physiological response to hypoxia and stress signals by direct up-regulation of inflammatory and pro-thrombotic genes. Moreover, a systems biology approach with human monocytes treated with the immunomodulatory peptide LL-37 revealed an involvement of Ap2, Sp1, E2f and Egr in gene regulation during conditions based on innate immunity [23]. Our data also predict that these transcription factors seem to be co-activated by various conditions of inflammation and synergize with well-known pro-inflammatory transcription factors such as NF-κB and AP-1 in a Rage-dependent manner. It is worth to note that TFBS for Sp1, Ap2, and Egr are also present in the promoter of *Rage *[24,25], suggesting a positive feedback loop by up-regulation of the receptor, which ensures maintenance and amplification of cellular activation in settings where ligands of Rage accumulate. A similar scenario has been described for NF-κB, which also represents a target of Rage signaling and activator of Rage expression [5,6].

Interestingly, our analysis revealed a significant enrichment of TFBS for the transcription factor E2f. There are a number of findings demonstrating diverse transcriptional regulation of E2f-responsive genes, suggesting that expression of these genes is regulated by different sets of Rb-E2f protein complexes [16]. However, it is currently uncertain how individual E2f members recognize a particular E2f-binding site during cell cycle progression or differentiation. One possibility is that the DNA-binding specificity of E2f members is influenced by other transcriptional regulatory factors, such as Sp1, that bind to sites contiguous to the E2f-binding site [26,27]. We found TPA-induced protein levels for E2f1 and E2f4 in epidermal keratinocytes of *wt *mice. At the same time, we observed a prominent up-regulation of Rb1 and Rbl1, suggesting the formation of Rb-E2f protein complexes. In contrast to *wt *mice, induction of E2f and Rb family proteins was impaired or only transient in keratinocytes of TPA-treated *Rage^-/- ^*back skin. Rb family proteins associate with a wide range of chromatin remodeling proteins forming transcriptional repressor complexes [28]. Thus, the existence of Rb-E2f complexes in keratinocytes after TPA stimulation could explain the enrichment of TFBS for E2f in the gene set characterized by strong and sustained repression. In addition to control of gene expression and binding to inhibitory Rb proteins, the activity of E2F proteins is tightly regulated by post-translational modification and regulation of protein turnover [29]. As an example, free E2F1 and E2F4 proteins are unstable due to ubiquitination and proteasomal degradation. Numerous cellular proteins have been described to regulate E2F protein ubiquitination, such as the CK1-MDM2 complex [30,31], ARF proteins (p14ARF in human and p19ARF in the mouse; [32]), Set9 and LSD1 [33]. However, our analysis did not reveal major changes in expression of any of these regulators upon TPA application or between RAGE-deficient mice and controls, and a functional link between RAGE signaling with one of these proteins has not been documented to the best of our knowledge. Ivanova and colleagues reported that in differentiating keratinocytes calcium-induced protein kinase C (PKC) activation reduces E2F1 protein level, which requires activation of novel PKC isoforms by the MAP kinase p38 [34]. Again E2F1 down-regulation in differentiating keratinocytes involves its ubiquitination and proteosomal degradation subsequent to CRM1-dependent nuclear export and degradation of E2F1 during differentiation [35]. Indeed, we observed strong cytoplasmic staining for E2F1 protein 12 hours after TPA treatment in keratinocytes of *Rage^-/- ^*mice and *wt *controls. However, in contrast to *wt *controls, which show obvious nuclear staining for E2F1 until 48 hours after treatment, nuclear staining in keratinocytes of *Rage^-/- ^*mice was hardly visible at any time point, suggesting that RAGE signaling might regulate nuclear-cytoplasmic shuttling of E2F proteins.

Finally, our data predict that the Rb-E2f pathway and its target genes not only act downstream of Rage signaling, but also might be pivotal for the process of skin inflammation upon TPA treatment. Indeed, CDK activity, which is up-stream of Rb-E2f, was recently correlated with roles in inflammatory cell differentiation, adhesion and recruitment as well as cytokine production and inflammatory signaling [36]. Intriguingly, CDK inhibitor drugs that are well-known to impair cell cycle progression in tumor cells have emerged recently as potential anti-inflammatory, pharmacological agents by influencing the resolution of inflammation [36,37].

## Conclusions

**Figure 5:**
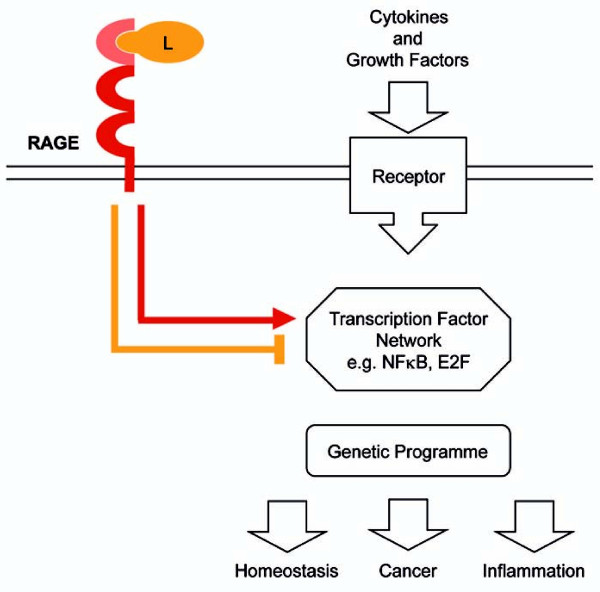
**Model of physiological and pathological functions of RAGE signaling**. Combination of gene expression profiling and computational approaches revealed that RAGE is not necessary for the initial response after stimulation, but absolute required for sustaining altered gene transcription. This is mainly due to the impact of RAGE-mediated signaling on expression and activity of transcription factors such as E2F. Thereby, RAGE modulates the kinetic of transcript levels of genes implicated in tissue homeostasis, inflammation, and cancer.

In summary, our approach to combine gene expression profiling with computational analysis did not only highlight the topology of Rage-dependent gene regulation in skin inflammation, but also allowed the prediction of novel transcription factors downstream of Rage signaling (Figure 5). A major challenge in the future will be the integration of known and newly identified transcription factors in a common model of Rage-dependent signaling network and to predict a dynamic program of inflammation in settings of physiological as well as pathological conditions.

## Methods

### Animal work and sample preparation

*Rage^-/- ^*animals were described previously [38], and *wt *controls were obtained from Charles River Laboratories. Mice were housed and treated with TPA as described previously [3]. In short, 10 nmol TPA/100 μl Acetone was applied to the shaved dorsal back skin and mice were sacrificed at indicated time points. Mice receiving acetone or no treatment served as controls. The procedures for performing animal experiments were in accordance with the principles and guidelines of the 'Arbeitsgemeinschaft der Tierschutzbeauftragten in Baden-Württemberg' and were approved by the 'Regierungspräsidium Karlsruhe', Germany (AZ 129/02).

Skin necropsies for RNA or protein preparation were immediately frozen in liquid nitrogen after isolation. For histological analysis, tissues were fixed with 4% paraformaldehyde (PFA) in PBS pH 7.4, paraffin embedded, and cut into 6 μm sections as described previously [3]. Tissue sections were stained with hematoxylin-eosin and were examined by several experienced experimenters.

### RNA preparation

Total RNA extraction from mouse back skin of untreated, 24 hours acetone- and 6, 12, 24, 48 hours TPA-treated *wt *and *Rage*^-/- ^mice was performed according to the manufacturer's instructions using peqGOLD RNAPure™ Reagent (Peq Lab, Erlangen, Germany). For RNA integrity and degradation analysis, the 2100 BioAnalyzer (Agilent Technologies, Santa Clara, CA) with RNA 6000 Nano LabChip Kit was used according to the manufacturer's instructions. Only RNA preparations with a RNA Integrity Number (RIN) of at least seven were used for microarray analysis.

### Microarray analysis

Global gene expression analysis was performed on 4x44K whole mouse genome one-color 60-mer oligonucleotide microarrays (Agilent Technologies) containing 41,174 unique probes. For amplification and single-color labeling of 1 μg RNA, the Low RNA Input Linear Amplification Kit and the One-Color RNA Spike-in Kit (Agilent Technologies, Santa Clara, CA) were used according to the manufacturer's protocol. Upon hybridization, microarray read out was accomplished in the Agilent Scanner G25505B (Agilent Technologies, Santa Clara, CA) with 5 μm resolution and automatically adjusting PMT voltages according to manufacturer's specification. Data processing was performed using Feature Extraction FE V9.5 Image Analysis software (Agilent Technologies, Santa Clara, CA) as recommended by the manufacturer.

### Quantitative real-time polymerase chain reaction analysis

Quantitative real-time polymerase chain reaction analysis was performed as described previously [3]. Primers used for RQ-PCR analysis are listed in Additional file 7. Target gene cycle of threshold values were normalized to the corresponding cycle of threshold values of using the change in cycle of threshold method.

### Statistical analysis

Array data were normalized by the quantile method [39], in combination with the "normexp" background correction implemented in Limma [40], and log2-transformed. Differentially expressed genes were identified by applying a linear model with the factors "time point" and "genotype", and subsequent empirical Bayesian correction [40]. For each time point *t*, the following contrast was calculated: (*I*_*r*,*t *_- *I*_*r*-0_) - (*I*_*w*,*t *_- *w*_*w*,0_), where *I *is the logarithm of the vector of intensities, indices *r *and *w *refer to *Rage^-/- ^*and *wt *mice, respectively, and 0 is the control condition (*t *= 0). *P*-values from the *F *test of the linear model were adjusted for multiple testing by the method of Benjamini and Hochberg [41]. All adjusted *P*-values < 0.05 were considered significant. All calculations were carried out in R version 2.6.2 http://www.R-project.org and Limma version 2.12.0. A list of differentially expressed genes at 24 hours after TPA stimulation is given in Additional file 3.

In order to further assess the dynamic response of the gene regulation upon TPA stimulation, we ranked the gene expression kinetics according to the peak and mean fold expression of each genes within the experimental time window of 48 hours [15]. Fold expression of each gene was calculated with respect to the control condition of *wt *and *Rage^-/- ^*animals. Next, a rank score *s *was defined for every gene as $s=\sqrt{FE_{t}^{2}+FE_{p}^{2}}$, where *FE_i _*= 〈*g_i_*(*t*)〉_*T *_and, *FE_p _*denote the time-averaged mean and peak gene fold expression (FE) of the gene's time series, respectively, normalized to the maximal peak and maximal mean fold expression of all measured genes. Gene ranking was performed separately for the three biological replicates of the TPA stimulation. Taking the 1,000 genes having the highest rank score *s *in each replicate, a set of 341 genes common to all rank lists was identified for further analysis (Additional file 1). Taking the top 500, 2,000 or 3,000 genes did not change the quality of the results. Subsequent k-means clustering of these 341 *wt *expression profiles was performed and expression levels of the same genes in *Rage^-/- ^*samples were superimposed. Pathway analysis was accomplished by using web-based DAVID [42,43].

### *In silico *promoter analysis

All available promoter sequences of murine genes represented on the whole mouse genome microarray were extracted from NCBI Entrezgene database ([44]; download: June 19th, 2008). Putative transcription factor binding sites (TFBS) were scanned within 2 kb upstream and downstream of the annotated transcriptional start site utilizing a position-weight matrix (PWM) scan as implemented in cureos package v0.3 ([45]; http://www.bepress.com/sagmb/vol2/iss1/art7) and described in Westermann et al. [46] for R open-source software http://www.R-project.org. PWMs were taken from the TRANSFAC database ([47]; release: January 12th, 2008). *P*-values for each PWM were obtained by comparing their scores to those of 1,000 random 4 kb sequence permutations. A general *P*-value cut-off of p < 0.1 was set as a reasonable compromise between false-positives and false-negatives. The genes that were differentially expressed between *wt *and *Rage^-/- ^*mice at the time point 24 hours after TPA application were divided into three sub-clusters with different gene expression profiles by unsupervised hierarchical clustering within samples at the time point t = 24 hours (complete linkage, the distance was calculated by one minus the Pearson correlation coefficient). Fisher's exact tests were performed to determine enrichments of PWM hits (counting genes with ≥ 1 significant score as a hit) for each cluster as compared to all other genes represented on the microarray. All *P*-values were Benjamini-Hochberg-corrected for multiple testing.

### Western Blot analysis

Western Blot analyses were performed with whole cell lysates from mouse back skin with antibodies listed in Additional file 8 according to the manufacturer's instructions. Whole cell lysates were prepared with RIPA buffer (50 mM Tris-HCL pH 8, 150 mM NaCl, 0.1% SDS, 0.5% deoxylacid Na^+^-salt, 1% NP-40).

### Immunohistochemistry analysis

Immunohistochemistry staining was performed on back skin sections from *wt *and *Rage^-/- ^*mice with the Immunodetection Kit (Vector Laboratories; Burlingame, CA) according to the manufacturer's instructions. Primary and secondary antibodies used are listed in Additional file 8.

## List of abbreviations

AGE: advanced glycation end products; AP1: Activator protein 1; AP2: Activator protein 2; CAC-bp: CAC-binding protein; E2f: E2-promoter-binding factor; Egr: Early growth response factor; Hmgb1: High mobility group box-1; JAK: Janus kinase; MAP kinases: mitogen-activated protein kinases; Maz: Myc-associated zinc-finger protein; Mazr: Myc-associated zinc-finger protein-related protein; Myc: v-myc meylocytomatosis viral oncogene homolog; NF-κB: Nuclear factor kappa B; Nr2f: nuclear receptor subfamily 2 group F members, PI3K: Phosphoinositide-3-kinase; PKC: protein kinase C; Rage: receptor for advanced glycation end products; Rb: Retinoblastoma protein; Rbl1: Retinoblastoma-like protein 1; Rbl2: Retinoblastoma-like protein 2; Sp1: Specificity protein 1; Sp4: Specificity protein 4; STAT: Signal transducer and activator of transcription; Tcfap2: transcription factor AP-2, alpha; TFBS: transcription factor binding sites; TPA: 12-O-tetradecanoylphorbol-13-acetate; Wt: wild-type; Wt1: Wilms tumor 1 homolog.

## Authors' contributions

AR, CG, PA and JH design and analysis of experimental research; AR, RM, JN and CG performed experimental research; TB, BB, HB, RK and RE designed and performed computational and statistical analysis; AB and PN provision of animal model system and analytic tools; AR and JH wrote the paper; TB, BB, HB, RM, JN, RK, CG, AB, PN, RE and PA critical review and editing of the manuscript.

None of the authors had any personal or financial conflicts of interest.

## Supplementary Material

Additional file 1 **Table with 341 TPA-responsive genes in back skin of *wt mice***. Click here for file

Additional file 2 **K-means clustering of TPA-responsive genes**. K-means clustering of common TPA-responsive genes in the kinetics of three independent experiments with *wt *animals revealed 6 clusters. Cluster 1 (n = 5), cluster 2 (n = 45), cluster 3 (n = 125), cluster 4 (n = 71), cluster 5 (n = 11), and cluster 6 (n = 84). Black lines represent transcript levels of genes in *wt *skin samples. Red lines represent transcript levels in *Rage^-/- ^*skin samples. Click here for file

Additional file 3 **Table of 122 genes differentially expressed 24 hours after TPA stimulation**. Click here for file

Additional file 4 **Quantitative real-time PCR of differentially expressed genes 24 hours after TPA treatment**. Relative transcript levels of differentially expressed genes were determined by quantitative real-time PCR with cDNA derived from *wt *and *Rage^-/- ^*back skin 24 hours upon TPA treatment. Transcript levels for genes of interest were determined in triplicates with *wt *and *Rage^-/- ^*cDNA samples and normalized to *Hprt *transcript levels. Next, expression values of genes of interest derived from *wt *cDNA were set to one and bars represent relative transcript levels for *Rage^-/- ^*cDNA samples. Similar data were obtained for two independent biological replicates (data not shown). Click here for file

Additional file 5 **Cluster dendrogram of genes differentially expressed at t = 24 hours**. Clustering was done only over samples from t = 24 hours via Person correlation distance, complete linkage algorithm. Three clusters were defined from the dendrogram. Click here for file

Additional file 6 **Rbl2 protein expression in skin following a single TPA stimulus**. Tissue sections of acetone- (Ac) or TPA-treated (6, 12, 24, and 48 hours) back skin from *wt *and *Rage^-/- ^*mice were analyzed by immunohistochemical staining using Rbl2-specific antibodies. Representative images of at least 2 animals of each genotype and time point are shown with red staining for Rbl2 and counterstaining with hematoxylin. Scale bar = 25 μm. Click here for file

Additional file 7 **Table of primer sequences used for quantitative real-time PCR analysis**. Click here for file

Additional file 8 **Table of primary antibodies used for Western Blot (WB) and immunohistochemistry analysis (IHC)**. Click here for file
