# Supplementary material for: Identification of the Rage-dependent gene regulatory network in a mouse model of skin inflammation
Source: BMC Genomics. 2010 Oct 5;11:537. doi: 10.1186/1471-2164-11-537 (PMC3091686; doi:10.1186/1471-2164-11-537)
Supplement: Additional file 1 — Table with 341 TPA-responsive genes in back skin of wt mice. [file 1471-2164-11-537-S1.DOC]

**Additional file 1: 341 TPA-responsive genes in back skin of *wt*** **mice** (C = k-means cluster)

|  |  |  |  | ***wt*** | | | | ***Rage^-/-^*** | | | |
| --- | --- | --- | --- | --- | --- | --- | --- | --- | --- | --- | --- |
| **C** | **Symbol** | **Description** | **Probe** | **6h** | **12h** | **24h** | **48h** | **6h** | **12h** | **24h** | **48h** |
| 1 | Krt1-10 | keratin complex 1 | A_52_P76277 | -0,17 | -2,01 | -1,88 | 0,22 | 0,42 | -1,07 | -0,90 | 0,68 |
| 1 | LOC433621 | similar to Acidic ribosomal phosphoprotein P0 | A_51_P200186 | -0,04 | -0,55 | 0,09 | 1,98 | -0,14 | -0,12 | 1,07 | 1,93 |
| 1 | Krt2-1 | keratin complex 2 | A_51_P416669 | -0,03 | -0,91 | 0,25 | 1,86 | 0,26 | 0,22 | 1,03 | 1,92 |
| 1 | Cyp2e1 | cytochrome P450 | A_51_P283456 | -0,09 | -1,81 | -1,07 | 0,13 | -0,38 | -1,17 | -0,76 | 0,08 |
| 1 | Krt1-10 | keratin complex 1 | A_51_P111285 | -0,03 | -1,69 | -1,56 | 0,18 | 0,33 | -0,87 | -0,78 | 0,47 |
| 2 | Slc4a7 | solute carrier family 4 | A_52_P108502 | -0,83 | -0,67 | -1,09 | -2,34 | 0,12 | 0,96 | 1,29 | 0,75 |
| 2 | 2310032F03Rik | RIKEN cDNA 2310032F03 gene | A_51_P391955 | -0,68 | -2,06 | -2,03 | -1,29 | 0,02 | -0,87 | -0,67 | -0,68 |
| 2 | Krt2-16 | keratin complex 2 | A_51_P367880 | -1,09 | -0,84 | -1,41 | -1,95 | -1,15 | -0,49 | -0,43 | -0,60 |
| 2 | 1810008K03Rik | RIKEN cDNA 1810008K03 gene | A_51_P134812 | -0,82 | -1,16 | -1,78 | -1,54 | -1,07 | -0,45 | -0,35 | -0,54 |
| 2 | Krt1-5 | keratin complex 1 | A_52_P545650 | -0,96 | -1,18 | -1,78 | -1,36 | -1,01 | -0,90 | -0,46 | -0,94 |
| 2 | Insig2 | insulin induced gene 2 | A_51_P439452 | -1,18 | -1,02 | -1,73 | -1,30 | -0,47 | -0,42 | -0,24 | -0,82 |
| 2 | P2ry5 | purinergic receptor P2Y | A_51_P234728 | -1,05 | -1,30 | -1,68 | -1,32 | -0,36 | -0,66 | -0,19 | -1,18 |
| 2 | 2310007B03Rik | RIKEN cDNA 2310007B03 gene | A_51_P275435 | -1,07 | -1,38 | -1,64 | -1,15 | -1,31 | -0,85 | -0,46 | -0,35 |
| 2 | Tm4sf4 | transmembrane 4 superfamily member 4 | A_51_P371001 | -0,80 | -0,78 | -1,28 | -1,78 | -0,73 | -0,39 | -0,44 | -0,53 |
| 2 | A_51_P358714 |  | A_51_P358714 | -0,95 | -1,16 | -1,62 | -1,31 | -0,17 | -0,43 | -0,35 | -1,00 |
| 2 | Krt1-5 | keratin complex 1 | A_51_P214275 | -0,90 | -1,15 | -1,62 | -1,31 | -0,97 | -0,93 | -0,37 | -0,88 |
| 2 | Slc25a37 | solute carrier family 25 | A_51_P343429 | -1,05 | -1,04 | -1,48 | -1,57 | -0,58 | -0,63 | -0,36 | -0,97 |
| 2 | BC038479 | cDNA sequence BC038479 | A_51_P104197 | -0,85 | -1,36 | -1,57 | -1,22 | -0,45 | -0,82 | -0,73 | -0,61 |
| 2 | Dio3 | deiodinase | A_52_P648524 | -1,10 | -0,94 | -1,34 | -1,58 | -0,68 | -0,74 | -0,37 | -0,81 |
| 2 | Calm2 | calmodulin 2 | A_51_P185971 | -0,77 | -1,09 | -1,61 | -1,30 | 0,37 | -0,35 | -0,19 | -1,15 |
| 2 | Tm4sf1 | transmembrane 4 superfamily member 1 | A_51_P240614 | -0,85 | -0,91 | -1,36 | -1,61 | 0,21 | -0,35 | -0,76 | -1,07 |
| 2 | Crot | carnitine O-octanoyltransferase | A_51_P489153 | -1,02 | -0,96 | -1,63 | -1,01 | -0,30 | -0,43 | -0,15 | -1,25 |
| 2 | Gm770 | gene model 770 | A_51_P242978 | -1,19 | -1,00 | -1,45 | -1,49 | -0,53 | -0,11 | -0,34 | -0,32 |
| 2 | Krtap3-1 | keratin associated protein 3-1 | A_52_P254286 | -0,90 | -1,10 | -1,54 | -1,35 | -1,19 | -0,61 | -0,23 | -0,63 |
| 2 | Gng13 | guanine nucleotide binding protein 13 | A_51_P149422 | -0,67 | -1,11 | -1,59 | -1,31 | -0,81 | -0,96 | -0,93 | -0,89 |
| 2 | Krt2-20 | keratin complex 2 | A_51_P239367 | -1,12 | -1,23 | -1,48 | -1,26 | -0,75 | -0,49 | 0,19 | -0,67 |
| 2 | Akr1c19 | aldo-keto reductase family 1 | A_51_P111335 | -1,11 | -1,41 | -1,43 | -1,31 | -0,63 | -0,40 | -0,79 | -0,41 |
| 2 | Krt1-18 | keratin complex 1 | A_51_P324814 | -0,60 | -0,68 | -1,34 | -1,66 | 0,03 | -0,22 | -0,30 | -0,90 |
| 2 | A030011M19 | hypothetical protein A030011M19 | A_51_P402175 | -1,04 | -1,08 | -1,49 | -1,31 | -1,08 | -0,70 | 0,04 | -0,75 |
| 2 | Pdzrn3 | PDZ domain containing RING finger 3 | A_52_P327176 | -1,12 | -1,25 | -1,43 | -1,32 | -0,49 | -0,50 | -0,15 | -0,92 |
| 2 | Dusp2 | dual specificity phosphatase 2 | A_52_P399934 | -0,86 | -0,79 | -1,33 | -1,57 | -0,34 | -0,39 | -0,22 | -0,89 |
| 2 | Stox1 | storkhead box 1 | A_52_P480470 | -1,12 | -1,05 | -1,48 | -1,24 | -0,69 | -0,50 | -0,26 | -0,55 |
| 2 | 9630015D15Rik | RIKEN cDNA 9630015D15 gene | A_51_P268953 | -0,85 | -0,95 | -1,52 | -1,27 | -0,29 | -0,68 | -0,45 | -1,07 |
| 2 | Tspan6 | tetraspanin 6 | A_51_P473272 | -0,95 | -1,17 | -1,36 | -1,42 | -0,67 | -0,76 | -0,56 | -1,02 |
| 2 | Fabp4 | fatty acid binding protein 4 | A_52_P564544 | -0,98 | -1,38 | -1,24 | -1,32 | -0,44 | -0,76 | -1,00 | -0,78 |
| 2 | Pik3r3 | phosphatidylinositol 3 kinase | A_52_P607103 | -1,20 | -0,88 | -1,44 | -1,20 | -0,83 | -0,47 | -0,40 | -1,07 |
| 2 | Gpr48 | G protein-coupled receptor 48 | A_52_P276727 | -0,90 | -1,05 | -1,45 | -1,24 | -0,34 | -0,44 | -0,44 | -1,07 |
| 2 | D11Ertd636e | DNA segment | A_51_P377557 | -0,98 | -1,15 | -1,39 | -1,28 | -1,15 | -0,58 | -0,50 | -0,39 |
| 2 | 9630015D15Rik | RIKEN cDNA 9630015D15 gene | A_52_P10017 | -0,84 | -0,75 | -1,54 | -1,05 | -0,38 | -0,43 | -0,15 | -0,77 |
| 2 | 4732454E20Rik | RIKEN cDNA 4732454E20 gene | A_51_P119882 | -0,92 | -1,05 | -1,31 | -1,40 | -0,62 | -0,36 | -0,19 | -0,94 |
| 2 | Thhl1 | trichohyalin-like 1 | A_51_P473912 | -1,16 | -0,99 | -1,37 | -1,21 | -0,32 | -0,28 | -0,02 | -0,37 |
| 2 | Slc9a2 | solute carrier family 9 (sodium/hydrogen exchanger) | A_51_P397983 | -0,79 | -0,97 | -1,42 | -1,35 | -0,56 | -0,57 | -0,32 | -0,72 |
| 2 | FHOS2 | formin-family protein FHOS2 | A_52_P326548 | -0,83 | -0,98 | -1,44 | -1,17 | -0,48 | -0,52 | -0,19 | -0,63 |
| 2 | A_52_P707535 |  | A_52_P707535 | -1,13 | -1,04 | -1,39 | -1,03 | -0,20 | -0,54 | -0,26 | -0,56 |
| 2 | Id4 | inhibitor of DNA binding 4 | A_51_P494430 | -1,14 | -1,22 | -1,37 | -0,86 | -0,87 | -0,63 | -0,25 | -0,65 |
| 2 | E330005K07Rik | RIKEN cDNA E330005K07 gene | A_52_P682456 | -0,83 | -1,03 | -1,40 | -1,07 | -0,12 | -0,18 | -0,21 | -0,61 |
| 2 | Amy1 | amylase 1 | A_51_P383638 | -0,77 | -1,11 | -1,41 | -1,01 | 0,64 | -0,29 | -0,49 | -0,69 |
| 2 | Plekhk1 | pleckstrin homology domain containing | A_51_P193832 | -1,33 | -0,95 | -1,27 | -1,00 | -1,15 | -0,46 | -0,17 | -0,49 |
| 2 | Tyrp1 | tyrosinase-related protein 1 | A_51_P431985 | -0,84 | -1,16 | -1,40 | -0,92 | -0,75 | -0,47 | -0,06 | -1,18 |
| 2 | Prkar2b | protein kinase | A_51_P221062 | -0,88 | -1,23 | -1,35 | -1,00 | 0,05 | -0,70 | -0,55 | -0,92 |
| 3 | A030004J04Rik | RIKEN cDNA A030004J04 gene | A_52_P160408 | -2,84 | -3,14 | -3,77 | -3,28 | -2,34 | -0,91 | -0,18 | -1,94 |
| 3 | Krtap6-1 | keratin associated protein 6-1 | A_52_P479051 | -2,98 | -3,27 | -3,48 | -3,57 | -2,79 | -0,45 | -0,33 | -1,91 |
| 3 | Krtap6-1 | keratin associated protein 6-1 | A_52_P225117 | -2,94 | -3,24 | -3,35 | -3,60 | -2,80 | -0,42 | -0,36 | -1,77 |
| 3 | Krtap6-1 | keratin associated protein 6-1 | A_51_P375360 | -2,99 | -3,10 | -3,40 | -3,57 | -2,22 | -0,75 | -0,69 | -2,06 |
| 3 | 2310015J09Rik | RIKEN cDNA 2310015J09 gene | A_51_P204350 | -2,73 | -3,31 | -3,65 | -3,05 | -2,51 | -0,57 | 0,27 | -1,75 |
| 3 | Krtap16-8 | keratin associated protein 16-8 | A_51_P104681 | -2,88 | -3,16 | -3,48 | -3,30 | -2,45 | -0,50 | -0,24 | -2,00 |
| 3 | Krtap8-1 | keratin associated protein 8-1 | A_51_P302324 | -2,71 | -3,10 | -3,50 | -3,26 | -2,36 | -0,47 | -0,01 | -1,78 |
| 3 | Krtap3-1 | keratin associated protein 3-1 | A_52_P321831 | -2,67 | -3,06 | -3,49 | -3,13 | -2,43 | -0,60 | 0,08 | -1,72 |
| 3 | Krtap16-7 | keratin associated protein 16-7 | A_51_P221823 | -2,61 | -2,95 | -3,49 | -3,27 | -2,28 | -0,51 | -0,35 | -1,80 |
| 3 | Krtap8-1 | keratin associated protein 8-1 | A_52_P131062 | -2,49 | -2,95 | -3,52 | -3,26 | -2,22 | -0,25 | -0,07 | -1,66 |
| 3 | 2300006N05Rik | RIKEN cDNA 2300006N05 gene | A_51_P120645 | -2,75 | -2,70 | -3,58 | -2,88 | -1,70 | -0,77 | -0,68 | -1,31 |
| 3 | Krtap16-9 | keratin associated protein 16-9 | A_52_P487979 | -2,56 | -3,08 | -3,17 | -3,39 | -2,53 | -0,31 | -0,10 | -2,18 |
| 3 | LOC435285 | similar to keratin associated protein 4-10 | A_51_P172424 | -2,71 | -2,92 | -3,42 | -3,03 | -2,25 | -0,62 | -0,08 | -1,71 |
| 3 | 1110032D16Rik | RIKEN cDNA 1110032D16 gene | A_51_P284503 | -2,74 | -3,01 | -3,36 | -3,15 | -2,24 | -0,33 | 0,09 | -1,73 |
| 3 | Krt1-4 | keratin complex 1 | A_51_P380991 | -2,71 | -3,07 | -3,29 | -3,12 | -2,39 | -0,59 | 0,20 | -1,70 |
| 3 | 4732407F15Rik | RIKEN cDNA 4732407F15 gene | A_51_P501873 | -2,50 | -2,74 | -3,39 | -3,11 | -1,65 | -0,50 | -0,18 | -1,61 |
| 3 | Krtap16-10 | keratin associated protein 16-10 | A_52_P463962 | -2,48 | -2,88 | -2,94 | -3,38 | -2,07 | -0,20 | 0,09 | -2,08 |
| 3 | Krtap6-1 | keratin associated protein 6-1 | A_51_P182876 | -2,75 | -2,92 | -3,14 | -3,21 | -2,26 | -0,55 | -0,32 | -1,81 |
| 3 | Krtap6-3 | keratin associated protein 6-3 | A_52_P2259 | -2,63 | -2,97 | -3,12 | -3,20 | -2,20 | -0,37 | -0,16 | -1,65 |
| 3 | Krt1-3 | keratin complex 1 | A_51_P336049 | -2,51 | -3,01 | -3,25 | -2,85 | -2,22 | -0,50 | 0,34 | -1,28 |
| 3 | AY026312 | cDNA sequence AY026312 | A_51_P386688 | -2,73 | -2,95 | -3,05 | -3,16 | -2,16 | -0,50 | 0,09 | -2,00 |
| 3 | Krtap3-1 | keratin associated protein 3-1 | A_51_P442723 | -2,48 | -2,76 | -3,18 | -2,97 | -1,92 | -0,66 | 0,02 | -1,60 |
| 3 | 4631426H08Rik | RIKEN cDNA 4631426H08 gene | A_51_P441898 | -2,46 | -3,06 | -3,23 | -2,41 | -2,36 | 0,01 | 0,72 | -1,02 |
| 3 | Krt25d | keratin 25D | A_51_P488780 | -2,19 | -2,82 | -3,32 | -2,48 | -1,91 | -0,53 | 0,06 | -1,53 |
| 3 | Krtap16-5 | keratin associated protein 16-5 | A_51_P345995 | -2,47 | -2,74 | -2,98 | -3,17 | -2,23 | -0,29 | 0,04 | -2,24 |
| 3 | Krt1-24 | keratin complex 1 | A_51_P107140 | -2,30 | -2,96 | -3,35 | -2,09 | -2,19 | -0,46 | 0,48 | -1,18 |
| 3 | Krtap14 | keratin associated protein 14 | A_51_P132400 | -2,40 | -2,53 | -3,09 | -3,17 | -1,88 | -0,47 | -0,10 | -1,77 |
| 3 | Krt2-6g | keratin complex 2 | A_51_P364639 | -2,53 | -3,10 | -3,17 | -2,26 | -2,74 | -0,11 | 0,58 | -0,90 |
| 3 | Car6 | carbonic anhydrase 6 | A_52_P310511 | -2,10 | -2,47 | -3,20 | -3,05 | -1,57 | -0,46 | -0,53 | -1,39 |
| 3 | Krt1-c29 | keratin complex-1 | A_51_P412926 | -2,51 | -3,12 | -3,09 | -2,41 | -2,49 | -0,50 | 0,50 | -1,16 |
| 3 | Krt1-1 | keratin complex 1 | A_51_P304200 | -2,34 | -2,70 | -3,19 | -2,63 | -1,98 | -0,71 | 0,22 | -1,50 |
| 3 | 2310043L02Rik | RIKEN cDNA 2310043L02 gene | A_51_P369773 | -2,35 | -2,67 | -3,04 | -3,07 | -1,73 | -0,53 | 0,11 | -1,59 |
| 3 | Crym | crystallin | A_51_P264695 | -2,20 | -2,50 | -3,14 | -2,79 | -1,71 | -0,78 | -0,36 | -1,59 |
| 3 | 1110033F04Rik | RIKEN cDNA 1110033F04 gene | A_51_P408199 | -2,41 | -2,46 | -3,06 | -2,86 | -1,69 | -0,74 | -0,16 | -1,43 |
| 3 | 2310040M23Rik | RIKEN cDNA 2310040M23 gene | A_51_P100624 | -2,49 | -2,64 | -2,95 | -2,94 | -1,82 | -0,49 | 0,03 | -1,56 |
| 3 | Krtap5-1 | keratin associated protein 5-1 | A_51_P305642 | -2,43 | -2,70 | -2,94 | -2,92 | -1,95 | -0,41 | 0,25 | -1,28 |
| 3 | Krtap16-1 | keratin associated protein 16-1 | A_51_P356695 | -2,28 | -2,45 | -2,67 | -3,07 | -2,12 | -0,34 | 0,13 | -1,98 |
| 3 | Krtap4-7 | keratin associated protein 4-7 | A_51_P335694 | -2,40 | -2,60 | -2,97 | -2,85 | -1,83 | -0,88 | -0,23 | -1,49 |
| 3 | A_52_P468068 |  | A_52_P468068 | -2,28 | -2,70 | -3,08 | -2,30 | -1,95 | -0,22 | 0,42 | -1,35 |
| 3 | A030006P16Rik | RIKEN cDNA A030006P16 gene | A_51_P370458 | -2,02 | -2,60 | -3,12 | -2,41 | -1,78 | -0,62 | 0,12 | -1,35 |
| 3 | Olfr1134 | olfactory receptor 1134 | A_51_P189899 | -2,22 | -2,40 | -2,89 | -2,99 | -1,65 | -0,49 | -0,63 | -1,37 |
| 3 | S100a3 | S100 calcium binding protein A3 | A_51_P468456 | -2,26 | -2,55 | -2,99 | -2,62 | -2,02 | -0,52 | 0,07 | -1,47 |
| 3 | Lrrc15 | leucine rich repeat containing 15 | A_52_P90805 | -2,22 | -2,75 | -3,06 | -2,13 | -1,72 | -0,98 | -0,38 | -1,38 |
| 3 | Krt2-10 | keratin complex 2 | A_52_P556448 | -2,15 | -2,49 | -3,00 | -2,62 | -1,89 | -0,68 | 0,37 | -1,22 |
| 3 | Krt2-25 | keratin complex 2 | A_52_P665240 | -2,20 | -2,41 | -2,98 | -2,63 | -1,87 | -0,74 | 0,32 | -1,33 |
| 3 | LOC432600 | similar to keratin associated protein 9-1 | A_51_P458722 | -2,21 | -2,44 | -2,92 | -2,61 | -1,43 | -0,70 | -0,40 | -1,44 |
| 3 | A030003K21Rik | RIKEN cDNA A030003K21 gene | A_51_P200034 | -2,29 | -2,63 | -2,86 | -2,62 | -1,77 | -0,57 | 0,11 | -1,36 |
| 3 | Krtap5-4 | keratin associated protein 5-4 | A_51_P504127 | -2,19 | -2,46 | -2,85 | -2,76 | -1,79 | -0,39 | 0,11 | -1,19 |
| 3 | Padi3 | peptidyl arginine deiminase | A_51_P450549 | -2,30 | -2,31 | -2,91 | -2,51 | -2,07 | -0,64 | -0,03 | -1,39 |
| 3 | A_52_P265712 |  | A_52_P265712 | -2,30 | -2,35 | -2,84 | -2,74 | -1,49 | -0,68 | -0,23 | -1,12 |
| 3 | Krtap3-3 | keratin associated protein 3-3 | A_51_P340481 | -2,17 | -2,43 | -2,86 | -2,67 | -1,87 | -0,34 | -0,01 | -1,62 |
| 3 | 5530401N06Rik | RIKEN cDNA 5530401N06 gene | A_51_P269721 | -2,27 | -2,50 | -2,84 | -2,54 | -1,53 | -0,46 | 0,11 | -1,41 |
| 3 | Krtap6-2 | keratin associated protein 6-2 | A_51_P350976 | -2,27 | -2,41 | -2,61 | -2,79 | -1,77 | -0,50 | -0,22 | -1,57 |
| 3 | A_51_P208722 |  | A_51_P208722 | -2,32 | -2,60 | -2,70 | -2,63 | -1,96 | -0,45 | 0,13 | -1,19 |
| 3 | Krtap3-2 | keratin associated protein 3-2 | A_52_P163660 | -1,46 | -2,38 | -2,89 | -2,76 | -1,26 | -0,82 | -0,45 | -1,47 |
| 3 | 2310002B14Rik | RIKEN cDNA 2310002B14 gene | A_51_P455861 | -2,09 | -2,24 | -2,79 | -2,62 | -1,59 | -0,78 | -0,14 | -1,38 |
| 3 | Krtap16-4 | keratin associated protein 16-4 | A_51_P360422 | -2,09 | -2,34 | -2,37 | -2,81 | -2,10 | -0,48 | 0,03 | -1,91 |
| 3 | Krtap13-1 | keratin associated protein 13-1 | A_51_P135118 | -2,01 | -2,32 | -2,71 | -2,37 | -1,70 | -0,32 | 0,42 | -1,47 |
| 3 | Krtap16-9 | keratin associated protein 16-9 | A_52_P487986 | -1,94 | -2,01 | -2,31 | -2,77 | -2,01 | -0,28 | -0,13 | -1,43 |
| 3 | A030005L19Rik | RIKEN cDNA A030005L19 gene | A_51_P241213 | -1,93 | -2,11 | -2,69 | -2,44 | -1,39 | -0,57 | -0,13 | -1,29 |
| 3 | Ly6g6d | lymphocyte antigen 6 complex | A_52_P12590 | -2,02 | -2,21 | -2,60 | -2,40 | -1,89 | -0,49 | -0,07 | -1,26 |
| 3 | A030014E15Rik | RIKEN cDNA A030014E15 gene | A_52_P1163599 | -1,95 | -2,26 | -2,53 | -2,34 | -1,72 | -0,56 | -0,14 | -1,37 |
| 3 | A_52_P238230 |  | A_52_P238230 | -2,24 | -2,41 | -2,21 | -2,17 | 0,10 | -0,12 | -0,18 | 0,08 |
| 3 | Krtap2-4 | keratin associated protein 2-4 | A_51_P475816 | -1,88 | -2,01 | -2,48 | -2,38 | -1,35 | -0,52 | 0,07 | -1,29 |
| 3 | A_52_P446985 |  | A_52_P446985 | -1,71 | -1,85 | -2,50 | -2,50 | -1,26 | -0,74 | -0,50 | -1,14 |
| 3 | Krtap9-1 | keratin associated protein 9-1 | A_51_P281673 | -1,71 | -1,74 | -2,39 | -2,44 | -1,55 | -0,84 | -0,54 | -0,97 |
| 3 | Ly6g6d | lymphocyte antigen 6 complex | A_51_P404246 | -1,82 | -1,98 | -2,34 | -2,21 | -1,52 | -0,42 | -0,13 | -1,04 |
| 3 | 4733401H21Rik | RIKEN cDNA 4733401H21 gene | A_51_P136680 | -1,57 | -1,70 | -1,92 | -2,51 | -1,06 | -0,64 | -0,28 | -1,13 |
| 3 | Gprc5d | G protein-coupled receptor | A_51_P433333 | -1,79 | -1,87 | -2,26 | -2,36 | -1,43 | -0,77 | -0,13 | -0,91 |
| 3 | Krtap12-1 | keratin associated protein 12-1 | A_51_P399789 | -1,92 | -1,84 | -2,35 | -2,21 | -1,40 | -0,47 | -0,08 | -0,85 |
| 3 | 4733401H21Rik | RIKEN cDNA 4733401H21 gene | A_52_P176993 | -1,75 | -1,90 | -2,18 | -2,38 | -1,36 | -0,54 | -0,21 | -1,15 |
| 3 | Otop2 | otopetrin 2 | A_51_P144222 | -1,66 | -2,10 | -2,35 | -2,12 | -1,32 | -0,77 | -0,48 | -1,16 |
| 3 | A030005K14Rik | RIKEN cDNA A030005K14 gene | A_51_P520224 | -1,96 | -1,90 | -2,28 | -2,14 | -1,20 | -0,53 | 0,10 | -1,01 |
| 3 | Krt2-18 | keratin complex 2 | A_51_P103396 | -1,83 | -1,97 | -2,36 | -1,84 | -1,48 | -0,78 | 0,37 | -1,01 |
| 3 | Krtap15 | keratin associated protein 15 | A_51_P498429 | -1,58 | -1,76 | -2,23 | -2,34 | -1,06 | -0,58 | 0,03 | -0,85 |
| 3 | Krtap16-10 | keratin associated protein 16-10 | A_52_P659514 | -1,71 | -1,90 | -2,18 | -2,23 | -1,44 | -0,44 | -0,34 | -0,87 |
| 3 | Mt4 | metallothionein 4 | A_51_P424275 | -1,03 | -1,84 | -2,48 | -1,60 | -1,40 | -1,03 | -0,80 | -1,00 |
| 3 | Sct | secretin | A_51_P234359 | -1,78 | -1,79 | -2,26 | -1,96 | -1,68 | -0,55 | -0,12 | -1,16 |
| 3 | A_51_P493437 |  | A_51_P493437 | -1,76 | -1,82 | -2,22 | -2,06 | -1,20 | -0,36 | 0,07 | -1,07 |
| 3 | Pck1 | phosphoenolpyruvate carboxykinase 1 | A_51_P250217 | -1,39 | -2,39 | -1,87 | -1,12 | -0,58 | -2,09 | -1,00 | -0,58 |
| 3 | Krt1-2 | keratin complex 1 | A_51_P360374 | -1,36 | -1,85 | -2,22 | -1,93 | -1,37 | -0,90 | -0,46 | -1,17 |
| 3 | Krtap8-2 | keratin associated protein 8-2 | A_51_P109412 | -1,67 | -1,65 | -1,98 | -2,16 | -1,03 | -0,18 | 0,19 | -0,96 |
| 3 | Eraf | erythroid associated factor | A_52_P534583 | -1,57 | -1,69 | -2,12 | -1,96 | -0,90 | -0,57 | -0,35 | -0,77 |
| 3 | Slc40a1 | solute carrier family 40 (iron-regulated transporter) | A_51_P389988 | -1,49 | -1,60 | -2,19 | -1,51 | -0,83 | -0,76 | -0,52 | -1,12 |
| 3 | AHF | hair follicle protein AHF | A_52_P200643 | -1,54 | -1,54 | -2,10 | -1,63 | -0,49 | -0,56 | 0,03 | -0,71 |
| 3 | Cryba4 | crystallin | A_51_P285299 | -1,39 | -1,48 | -2,11 | -1,80 | -1,45 | -0,78 | -0,20 | -1,31 |
| 3 | Mt4 | metallothionein 4 | A_51_P424272 | -0,96 | -1,74 | -2,17 | -1,58 | -1,26 | -1,07 | -0,89 | -1,00 |
| 3 | AI646023 | expressed sequence AI646023 | A_52_P151393 | -1,68 | -1,70 | -1,97 | -1,72 | -1,31 | -0,50 | -0,18 | -1,18 |
| 3 | Hamp2 | hepcidin antimicrobial peptide 2 | A_52_P21486 | -0,57 | -1,52 | -2,02 | -2,17 | 0,91 | -0,23 | -1,28 | -0,69 |
| 3 | Mlana | melan-A | A_51_P499623 | -1,29 | -1,94 | -2,03 | -1,37 | -1,46 | -0,77 | -0,40 | -1,13 |
| 3 | LOC546508 | similar to keratin associated protein 2-4 | A_52_P519317 | -1,32 | -1,40 | -1,91 | -2,01 | -1,07 | -0,63 | -0,31 | -0,83 |
| 3 | Trpv6 | transient receptor potential cation channel | A_51_P479029 | -1,42 | -1,52 | -2,01 | -1,54 | -0,93 | -0,77 | -0,63 | -0,63 |
| 3 | Xrcc1 | X-ray repair complementing defective repair in Chinese hamster cells 1 | A_51_P259694 | -1,75 | -1,63 | -1,88 | -1,63 | -1,69 | -0,46 | -0,09 | -0,53 |
| 3 | 2310061N02Rik | RIKEN cDNA 2310061N02 gene | A_51_P442765 | -1,31 | -1,35 | -1,97 | -1,90 | -0,83 | -0,40 | -0,08 | -0,77 |
| 3 | Trpm1 | transient receptor potential cation channel | A_51_P338031 | -1,40 | -1,75 | -1,93 | -1,52 | -1,25 | -0,38 | 0,01 | -1,11 |
| 3 | Igj | immunoglobulin joining chain | A_51_P150710 | -1,63 | -1,80 | -1,77 | -1,74 | 0,07 | 0,09 | -0,02 | -0,12 |
| 3 | 9230117E20Rik | RIKEN cDNA 9230117E20 gene | A_51_P370552 | -1,93 | -1,54 | -1,43 | -1,57 | -0,82 | -0,20 | -0,10 | -0,46 |
| 3 | Kcne1 | potassium voltage-gated channel | A_51_P211671 | -1,45 | -1,33 | -1,85 | -1,88 | -1,10 | -0,70 | -0,36 | -0,80 |
| 3 | A_52_P124734 |  | A_52_P124734 | -1,22 | -1,56 | -1,89 | -1,66 | -0,93 | -0,72 | -0,26 | -1,07 |
| 3 | Gabrp | gamma-aminobutyric acid (GABA-A) receptor | A_52_P184609 | -1,34 | -1,53 | -1,86 | -1,66 | -0,92 | -0,87 | -0,58 | -1,35 |
| 3 | Slc38a5 | solute carrier family 38 | A_51_P400269 | -1,73 | -1,76 | -1,47 | -1,73 | -0,36 | -0,46 | -0,43 | -0,40 |
| 3 | Igh-6 | immunoglobulin heavy chain 6 (heavy chain of IgM) | A_52_P571419 | -1,56 | -1,94 | -1,51 | -1,04 | -0,22 | -0,59 | -0,56 | 0,11 |
| 3 | Capn8 | calpain 8 | A_52_P530620 | -1,46 | -1,41 | -1,87 | -1,56 | -0,94 | -0,49 | -0,20 | -0,85 |
| 3 | A830053O21Rik | RIKEN cDNA A830053O21 gene | A_51_P107463 | -1,14 | -1,44 | -1,86 | -1,58 | -1,21 | -0,66 | -0,14 | -1,08 |
| 3 | Xrcc1 | X-ray repair complementing defective repair in Chinese hamster cells 1 | A_51_P259689 | -1,57 | -1,52 | -1,75 | -1,52 | -1,51 | -0,65 | -0,24 | -0,50 |
| 3 | Serpina1d | serine (or cysteine) proteinase inhibitor | A_51_P236303 | -1,39 | -1,51 | -1,70 | -1,73 | -1,12 | -0,77 | -0,35 | -0,66 |
| 3 | Bcr | breakpoint cluster region homolog | A_51_P290974 | -1,23 | -1,22 | -1,70 | -1,80 | -1,05 | -0,57 | -0,31 | -0,92 |
| 3 | 1110032A04Rik | RIKEN cDNA 1110032A04 gene | A_52_P649276 | -1,33 | -1,48 | -1,42 | -1,73 | -0,86 | -0,41 | -0,11 | -0,73 |
| 3 | Msx2 | homeo box | A_51_P388661 | -1,24 | -1,35 | -1,74 | -1,40 | -1,31 | -0,42 | -0,11 | -0,79 |
| 3 | Fgf5 | fibroblast growth factor 5 | A_52_P569375 | -1,56 | -1,36 | -1,53 | -1,64 | -1,18 | -0,55 | -0,32 | -1,04 |
| 3 | Fbp1 | fructose bisphosphatase 1 | A_51_P474701 | -1,53 | -0,97 | -1,56 | -1,72 | -1,66 | -0,28 | -0,01 | -1,12 |
| 3 | A_51_P284125 |  | A_51_P284125 | -1,27 | -1,11 | -1,46 | -1,75 | -0,66 | -0,63 | -0,65 | -0,65 |
| 3 | S100a15 | S100 calcium binding protein A15 | A_52_P63855 | -1,43 | -1,27 | -1,08 | -1,75 | -0,76 | -0,45 | 0,01 | -0,75 |
| 3 | Car3 | carbonic anhydrase 3 | A_52_P639461 | -1,69 | -1,58 | -1,50 | -0,98 | -0,31 | -0,67 | -0,38 | -0,83 |
| 3 | Crisp1 | cysteine-rich secretory protein 1 | A_52_P370268 | -1,21 | -1,31 | -1,54 | -1,68 | -0,75 | -0,60 | -0,64 | -1,29 |
| 3 | LOC545847 | similar to anti-colorectal carcinoma light chain | A_51_P390937 | -1,52 | -1,46 | -1,56 | -1,42 | 0,10 | 0,22 | 0,04 | 0,22 |
| 3 | LOC546508 | similar to keratin associated protein 2-4 | A_52_P519324 | -1,20 | -1,23 | -1,61 | -1,58 | -0,92 | -0,59 | -0,15 | -0,42 |
| 3 | Gm312 | gene model 312 | A_52_P641641 | -1,14 | -1,13 | -1,64 | -1,55 | -0,85 | -0,48 | -0,38 | -1,00 |
| 3 | Bambi | BMP and activin membrane-bound inhibitor | A_51_P248819 | -1,10 | -1,31 | -1,60 | -1,46 | -1,13 | -0,82 | -0,48 | -1,08 |
| 3 | 5430433J05Rik | RIKEN cDNA 5430433J05 gene | A_51_P144270 | -1,16 | -1,12 | -1,56 | -1,61 | -0,77 | -0,62 | -0,26 | -0,74 |
| 3 | Slc6a19 | solute carrier family 6 (neurotransmitter transporter) | A_51_P371214 | -1,12 | -1,39 | -1,63 | -1,19 | -1,19 | -0,84 | -0,80 | -0,58 |
| 3 | A_52_P17582 |  | A_52_P17582 | -1,17 | -1,01 | -1,61 | -1,46 | -0,72 | -0,39 | -0,05 | -0,58 |
| 3 | Ctse | cathepsin E | A_51_P217906 | -1,24 | -1,25 | -1,49 | -1,53 | -0,88 | -0,59 | -0,27 | -0,81 |
| 3 | D730001G18Rik | RIKEN cDNA D730001G18 gene | A_51_P222664 | -1,12 | -1,34 | -1,56 | -1,30 | -0,99 | -0,62 | -0,10 | -0,63 |
| 3 | Igj | immunoglobulin joining chain | A_51_P150705 | -1,38 | -1,33 | -1,29 | -1,24 | 0,05 | -0,03 | -0,06 | -0,11 |
| 4 | Sprr2d | small proline-rich protein 2D | A_51_P435588 | 3,49 | 4,85 | 4,81 | 4,01 | 2,63 | 3,98 | 3,59 | 2,75 |
| 4 | Sprr2a | small proline-rich protein 2A | A_52_P18887 | 2,56 | 3,49 | 3,96 | 4,42 | 2,03 | 3,59 | 3,70 | 3,89 |
| 4 | 2310002A05Rik | RIKEN cDNA 2310002A05 gene | A_52_P40244 | 2,75 | 3,54 | 3,81 | 3,13 | 0,64 | 2,46 | 2,28 | 2,33 |
| 4 | Saa3 | serum amyloid A 3 | A_51_P337308 | 2,25 | 3,51 | 3,95 | 2,43 | 1,05 | 2,09 | 1,95 | 1,77 |
| 4 | Slpi | secretory leukocyte protease inhibitor | A_52_P472324 | 2,74 | 3,63 | 3,28 | 2,28 | 2,54 | 3,03 | 2,26 | 1,30 |
| 4 | Ccl4 | chemokine (C-C motif) ligand 4 | A_51_P509573 | 2,98 | 3,52 | 3,21 | 1,37 | 3,43 | 2,94 | 1,87 | 0,68 |
| 4 | Saa2 | serum amyloid A 2 | A_51_P166886 | 1,93 | 3,40 | 3,46 | 1,57 | 1,48 | 2,16 | 2,13 | 0,85 |
| 4 | Sprr2f | small proline-rich protein 2F | A_51_P386870 | 1,69 | 3,54 | 3,18 | 1,18 | 1,73 | 3,17 | 2,39 | 0,52 |
| 4 | Stfa2 | stefin A2 | A_51_P135944 | 2,11 | 2,74 | 2,99 | 3,09 | 1,72 | 2,73 | 2,55 | 2,32 |
| 4 | Saa1 | serum amyloid A 1 | A_52_P318673 | 1,64 | 2,91 | 3,25 | 1,76 | 0,97 | 1,73 | 1,79 | 1,35 |
| 4 | Saa1 | serum amyloid A 1 | A_51_P361650 | 1,54 | 2,98 | 3,18 | 1,38 | 1,36 | 1,90 | 1,92 | 0,62 |
| 4 | Stfa3 | stefin A3 | A_51_P504815 | 1,89 | 2,31 | 2,58 | 2,94 | 1,18 | 2,11 | 1,95 | 2,00 |
| 4 | Timp1 | tissue inhibitor of metalloproteinase 1 | A_52_P87713 | 2,23 | 2,93 | 2,63 | 1,28 | 2,29 | 2,33 | 1,40 | 0,37 |
| 4 | Pigt | phosphatidylinositol glycan | A_51_P338803 | 2,27 | 1,72 | 2,40 | 2,79 | 2,27 | -0,01 | 0,56 | 1,70 |
| 4 | Sprr2e | small proline-rich protein 2E | A_51_P355014 | 1,61 | 2,63 | 2,66 | 2,61 | 1,10 | 2,76 | 2,61 | 2,56 |
| 4 | S100a9 | S100 calcium binding protein A9 (calgranulin B) | A_51_P402943 | 1,80 | 2,75 | 2,73 | 1,80 | 3,04 | 2,61 | 2,31 | 1,12 |
| 4 | Sprr2g | small proline-rich protein 2G | A_51_P516016 | 1,78 | 2,67 | 2,57 | 1,47 | 0,74 | 2,08 | 1,47 | 0,97 |
| 4 | Sprrl1 | small proline rich-like 1 | A_51_P373393 | 1,82 | 2,61 | 2,30 | 1,78 | 0,16 | 1,95 | 0,97 | 0,85 |
| 4 | Lpo | lactoperoxidase | A_51_P175667 | 1,75 | 2,45 | 2,33 | 2,30 | 0,90 | 1,85 | 1,56 | 1,52 |
| 4 | Lcn2 | lipocalin 2 | A_51_P510156 | 1,34 | 2,57 | 2,70 | 1,16 | 1,46 | 1,77 | 1,74 | 0,54 |
| 4 | Sprrl1 | small proline rich-like 1 | A_51_P373390 | 1,77 | 2,52 | 2,18 | 1,70 | 0,13 | 1,88 | 0,85 | 0,83 |
| 4 | Stfa1 | stefin A1 | A_51_P132185 | 1,49 | 2,28 | 2,42 | 2,27 | 1,44 | 2,09 | 2,07 | 1,45 |
| 4 | Chi3l1 | chitinase 3-like 1 | A_51_P362066 | 1,20 | 2,34 | 2,48 | 1,78 | 0,89 | 2,03 | 2,03 | 1,34 |
| 4 | Sprr2b | small proline-rich protein 2B | A_51_P459894 | 1,37 | 2,25 | 2,30 | 2,13 | 0,79 | 2,32 | 2,16 | 2,16 |
| 4 | Prss27 | protease | A_51_P263568 | 1,67 | 2,28 | 2,23 | 1,79 | 1,17 | 1,87 | 1,44 | 1,37 |
| 4 | Sprr1b | small proline-rich protein 1B | A_51_P508530 | 1,55 | 1,69 | 2,31 | 2,28 | -0,23 | 1,46 | 1,40 | 1,58 |
| 4 | Defb14 | defensin beta 14 | A_52_P68883 | 1,45 | 1,92 | 2,34 | 1,31 | 0,45 | 1,85 | 1,23 | 1,17 |
| 4 | Sprr2j | small proline-rich protein 2J | A_51_P491758 | 1,22 | 2,21 | 2,23 | 1,81 | 0,74 | 2,29 | 2,09 | 2,05 |
| 4 | LOC433016 | similar to cystatin A | A_51_P401907 | 1,28 | 2,15 | 2,37 | 1,11 | 2,36 | 2,20 | 1,40 | 0,46 |
| 4 | Gm566 | gene model 566 | A_51_P309307 | 1,33 | 2,07 | 2,22 | 1,58 | 1,58 | 2,31 | 2,10 | 1,25 |
| 4 | Serpina3n | serine (or cysteine) proteinase inhibitor | A_51_P159453 | 1,49 | 1,95 | 2,22 | 1,32 | 1,57 | 1,32 | 1,35 | 1,06 |
| 4 | Prss18 | protease | A_51_P258570 | 0,79 | 1,87 | 2,28 | 1,81 | 0,24 | 1,76 | 1,92 | 1,65 |
| 4 | A_51_P445823 |  | A_51_P445823 | 1,33 | 1,87 | 2,11 | 1,91 | 0,44 | 1,41 | 1,23 | 1,47 |
| 4 | Krt2-6b | keratin complex 2 | A_51_P126275 | 0,93 | 1,73 | 2,25 | 1,61 | 0,66 | 1,41 | 1,63 | 1,23 |
| 4 | MGI:3524944 | stefin A2 like 1 | A_52_P398925 | 1,15 | 1,78 | 1,65 | 2,19 | 1,50 | 2,02 | 1,52 | 1,33 |
| 4 | Mlze | melanoma-derived leucine zipper | A_51_P150489 | 0,84 | 1,58 | 1,61 | 2,26 | 0,81 | 1,95 | 2,17 | 2,01 |
| 4 | Sprr2k | small proline-rich protein 2K | A_51_P411316 | 1,03 | 1,97 | 2,06 | 1,86 | 0,66 | 2,13 | 2,02 | 1,99 |
| 4 | Lars2 | leucyl-tRNA synthetase | A_51_P314501 | 1,76 | 1,11 | 1,74 | 2,10 | 1,91 | -0,33 | 0,64 | 1,85 |
| 4 | Chga | chromogranin A | A_51_P358316 | 1,20 | 1,96 | 1,40 | 2,08 | -0,02 | 1,35 | 1,36 | 1,79 |
| 4 | 2310026J01Rik | RIKEN cDNA 2310026J01 gene | A_52_P203948 | 1,53 | 1,81 | 1,92 | 1,85 | 1,04 | 1,36 | 1,31 | 1,21 |
| 4 | Ankk1 | ankyrin repeat and kinase domain containing 1 | A_51_P158545 | 0,55 | 1,82 | 2,17 | 1,48 | 0,25 | 1,19 | 1,17 | 0,95 |
| 4 | Krt1-16 | keratin complex 1 | A_51_P457747 | 1,32 | 1,83 | 1,89 | 1,69 | 1,40 | 2,01 | 1,92 | 1,64 |
| 4 | A_51_P507669 |  | A_51_P507669 | 1,54 | 1,41 | 1,62 | 1,87 | 0,73 | -0,65 | -0,63 | 1,12 |
| 4 | Il1f6 | interleukin 1 family | A_51_P504194 | 1,00 | 1,31 | 1,61 | 1,96 | 0,31 | 1,14 | 1,14 | 1,51 |
| 4 | Degs2 | degenerative spermatocyte homolog 2 (Drosophila) | A_51_P122649 | 0,82 | 1,63 | 1,86 | 1,77 | 0,03 | 1,66 | 1,73 | 1,64 |
| 4 | Rptn | repetin | A_52_P523146 | 0,02 | 0,57 | 0,70 | 2,20 | 0,45 | 0,72 | 0,86 | 1,39 |
| 4 | 1110058A15Rik | RIKEN cDNA 1110058A15 gene | A_52_P630463 | 0,91 | 0,92 | 1,66 | 1,84 | -0,46 | 0,75 | 1,03 | 1,54 |
| 4 | Gsta4 | glutathione S-transferase | A_51_P112223 | 0,93 | 1,01 | 1,86 | 0,98 | 0,45 | 1,28 | 1,23 | 0,55 |
| 4 | Tcf23 | transcription factor 23 | A_52_P300451 | 0,52 | 0,45 | 1,40 | 1,95 | -0,09 | 0,35 | 0,84 | 1,41 |
| 4 | 1200016C12Rik | RIKEN cDNA 1200016C12 gene | A_51_P417956 | 1,00 | 1,47 | 1,66 | 1,36 | 0,18 | 1,19 | 1,10 | 1,36 |
| 4 | Prss19 | protease | A_52_P216672 | 0,72 | 1,18 | 1,68 | 1,70 | -0,30 | 0,91 | 1,08 | 1,29 |
| 4 | Myl7 | myosin | A_51_P246345 | 0,29 | 1,45 | 1,79 | 0,96 | -0,11 | 0,68 | 0,80 | 0,60 |
| 4 | Ptges | prostaglandin E synthase | A_51_P312327 | 1,32 | 1,53 | 1,45 | 1,09 | 1,25 | 1,26 | 0,96 | 0,83 |
| 4 | 1110031B11Rik | RIKEN cDNA 1110031B11 gene | A_51_P314465 | 0,82 | 0,89 | 1,45 | 1,64 | -0,25 | 0,73 | 0,94 | 1,38 |
| 4 | AJ430384 | cDNA sequence AJ430384 | A_51_P126327 | 1,04 | 1,10 | 1,46 | 1,55 | 0,37 | 0,98 | 1,17 | 1,16 |
| 4 | Aurkc | aurora kinase C | A_51_P512293 | 0,98 | 1,03 | 1,56 | 1,24 | -0,09 | 0,80 | 0,48 | 1,02 |
| 4 | Krt2-6a | keratin complex 2 | A_52_P104658 | 0,62 | 1,28 | 1,60 | 0,97 | 0,48 | 1,16 | 1,41 | 0,80 |
| 4 | Rbp2 | retinol binding protein 2 | A_51_P399143 | 0,53 | 1,21 | 1,53 | 1,48 | -0,07 | 1,23 | 1,25 | 1,29 |
| 4 | LOC434481 | similar to hypothetical protein 1 (rRNA external transcribed spacer) - mouse | A_52_P115191 | 0,81 | 0,88 | 1,17 | 1,59 | 0,21 | -0,10 | 0,34 | 0,82 |
| 4 | Smpd3 | sphingomyelin phosphodiesterase 3 | A_52_P478025 | 0,70 | 1,13 | 1,52 | 1,35 | -0,16 | 0,71 | 0,86 | 1,25 |
| 4 | Cxcl16 | chemokine (C-X-C motif) ligand 16 | A_51_P374203 | 1,25 | 1,30 | 1,39 | 1,23 | 0,76 | 0,92 | 0,82 | 1,09 |
| 4 | Stfa1 | stefin A1 | A_52_P487686 | 0,73 | 1,34 | 1,48 | 1,22 | 0,77 | 1,25 | 1,21 | 0,76 |
| 4 | Rbp2 | retinol binding protein 2 | A_52_P125363 | 0,52 | 1,16 | 1,50 | 1,45 | -0,10 | 1,12 | 1,09 | 1,13 |
| 4 | Hrnr | hornerin | A_51_P248986 | 0,52 | 1,05 | 0,77 | 1,62 | 0,17 | 0,86 | 0,73 | 0,53 |
| 4 | Slc16a3 | solute carrier family 16 (monocarboxylic acid transporters) | A_51_P144090 | 0,88 | 1,30 | 1,48 | 0,85 | 1,05 | 1,49 | 1,25 | 0,96 |
| 4 | Egln3 | EGL nine homolog 3 (C. elegans) | A_52_P387009 | 0,85 | 1,38 | 1,25 | 1,39 | 0,99 | 1,42 | 1,65 | 1,32 |
| 4 | A_51_P333594 |  | A_51_P333594 | 0,87 | 0,93 | 1,47 | 1,25 | 0,10 | 0,84 | 0,66 | 1,12 |
| 4 | Arc | activity regulated cytoskeletal-associated protein | A_51_P503494 | 1,43 | 1,11 | 1,15 | 0,96 | 1,21 | 0,93 | 0,50 | 0,58 |
| 4 | H19 | H19 fetal liver mRNA | A_51_P142196 | 1,16 | 0,95 | 1,30 | 1,38 | 0,60 | 0,14 | 0,37 | 1,08 |
| 4 | 4732465J04Rik | RIKEN cDNA 4732465J04 gene | A_52_P489202 | 1,16 | 1,20 | 1,35 | 1,15 | 0,62 | 1,09 | 0,48 | 0,66 |
| 4 | Cdk5r2 | cyclin-dependent kinase 5 | A_51_P189803 | 0,82 | 0,35 | 1,11 | 1,58 | 0,56 | -0,31 | 0,16 | 1,20 |
| 5 | Dbp | D site albumin promoter binding protein | A_51_P180492 | -1,21 | -2,35 | -0,57 | -0,44 | -1,01 | -2,34 | -0,75 | -0,03 |
| 5 | Dct | dopachrome tautomerase | A_51_P116838 | -0,84 | -1,85 | -1,58 | -0,83 | -0,91 | -0,66 | -0,21 | -0,88 |
| 5 | Mmp11 | matrix metalloproteinase 11 | A_51_P293087 | -1,57 | -1,31 | -0,88 | -0,54 | -0,94 | -0,56 | -0,80 | 0,08 |
| 5 | Adipoq | adiponectin | A_51_P458451 | -0,65 | -1,49 | -1,37 | -0,91 | 0,06 | -0,74 | -0,99 | -0,30 |
| 5 | Bbox1 | butyrobetaine (gamma) | A_51_P446570 | -0,82 | -1,42 | -1,45 | -0,74 | -0,15 | -0,72 | -0,37 | -0,13 |
| 5 | Si | silver | A_51_P208145 | -0,85 | -1,45 | -1,49 | -0,46 | -1,07 | -0,60 | -0,01 | -0,61 |
| 5 | Lrrc17 | leucine rich repeat containing 17 | A_51_P301930 | -0,84 | -1,62 | -0,67 | -0,19 | -0,57 | -1,08 | -0,62 | -0,30 |
| 5 | Hlf | hepatic leukemia factor | A_51_P134228 | -0,87 | -1,48 | -1,12 | -0,58 | -0,60 | -1,13 | -0,82 | -0,21 |
| 5 | LOC433620 | hypothetical gene supported by AK036878 | A_52_P1035965 | -0,51 | -1,39 | -1,48 | -0,63 | 0,09 | -0,40 | -0,29 | -0,24 |
| 5 | A_52_P1011632 |  | A_52_P1011632 | -0,69 | -1,29 | -1,49 | -0,44 | -0,12 | -0,97 | -0,73 | -0,11 |
| 5 | Retnla | resistin like alpha | A_52_P254817 | -0,53 | -1,09 | -1,56 | -0,40 | -0,02 | -1,03 | -1,10 | -0,13 |
| 6 | Timp1 | tissue inhibitor of metalloproteinase 1 | A_51_P512384 | 1,94 | 2,53 | 2,36 | 0,97 | 2,12 | 1,84 | 1,00 | 0,38 |
| 6 | Cxcl1 | chemokine (C-X-C motif) ligand 1 | A_51_P363187 | 2,40 | 2,21 | 2,00 | 0,55 | 3,04 | 2,02 | 0,85 | -0,28 |
| 6 | Ptx3 | pentraxin related gene | A_52_P26161 | 2,15 | 2,47 | 2,08 | 0,12 | 3,39 | 2,04 | 1,01 | 0,03 |
| 6 | Cxcl5 | chemokine (C-X-C motif) ligand 5 | A_52_P295432 | 1,78 | 2,52 | 1,83 | 0,12 | 2,47 | 2,54 | 0,92 | -0,04 |
| 6 | Il1rl1 | interleukin 1 receptor-like 1 | A_51_P339793 | 1,67 | 2,33 | 2,08 | 0,63 | 0,99 | 1,67 | 0,34 | -0,64 |
| 6 | Cd300lf | CD300 antigen like family member F | A_52_P422494 | 1,60 | 2,24 | 2,30 | 0,71 | 2,72 | 2,45 | 1,66 | 0,52 |
| 6 | Timp1 | tissue inhibitor of metalloproteinase 1 | A_51_P512379 | 1,71 | 2,27 | 2,13 | 0,87 | 1,98 | 1,73 | 0,97 | 0,30 |
| 6 | Ptx3 | pentraxin related gene | A_51_P374726 | 2,08 | 2,36 | 1,92 | 0,02 | 3,47 | 2,17 | 1,07 | 0,24 |
| 6 | Nppb | natriuretic peptide precursor type B | A_51_P426195 | 1,66 | 2,22 | 2,24 | 0,64 | 1,73 | 2,14 | 1,30 | 0,37 |
| 6 | Gm1960 | gene model 1960 | A_52_P232813 | 1,84 | 2,26 | 2,05 | 0,46 | 2,82 | 2,68 | 1,47 | 0,26 |
| 6 | Il19 | interleukin 19 | A_51_P142703 | 2,00 | 1,95 | 2,05 | 0,92 | 2,92 | 2,11 | 1,49 | 0,49 |
| 6 | Prg4 | proteoglycan 4 (megakaryocyte stimulating factor | A_51_P280455 | 1,22 | 2,02 | 2,18 | 0,92 | 1,75 | 1,99 | 1,71 | 0,99 |
| 6 | Retnlg | resistin like gamma | A_52_P425839 | 1,07 | 2,20 | 2,25 | 0,54 | 1,94 | 1,94 | 0,94 | 0,02 |
| 6 | LOC547372 | similar to LRRGT00049 | A_52_P615051 | 2,12 | 2,13 | 1,40 | 0,91 | 2,70 | 1,78 | 1,35 | 0,32 |
| 6 | Rrad | Ras-related associated with diabetes | A_51_P240253 | 1,80 | 2,38 | 1,03 | 0,18 | 2,43 | 1,79 | 0,76 | 0,42 |
| 6 | Csf3r | colony stimulating factor 3 receptor (granulocyte) | A_51_P515639 | 1,20 | 1,88 | 2,17 | 1,03 | 1,57 | 1,61 | 1,11 | 0,58 |
| 6 | Mmp9 | matrix metalloproteinase 9 | A_51_P401797 | 0,73 | 1,61 | 2,24 | 0,91 | 0,72 | 1,19 | 1,04 | 0,72 |
| 6 | A_52_P49797 |  | A_52_P49797 | 2,05 | 2,01 | 1,33 | 0,88 | 2,33 | 1,45 | 1,04 | 0,52 |
| 6 | Hdc | histidine decarboxylase | A_51_P254656 | 1,79 | 1,83 | 1,99 | 0,77 | 2,40 | 1,76 | 0,93 | 0,41 |
| 6 | Ccl3 | chemokine (C-C motif) ligand 3 | A_51_P140710 | 1,71 | 1,98 | 1,92 | 0,75 | 2,43 | 1,91 | 1,21 | 0,14 |
| 6 | Mmp9 | matrix metalloproteinase 9 | A_52_P507214 | 0,70 | 1,46 | 2,22 | 0,79 | 0,64 | 1,16 | 1,20 | 0,64 |
| 6 | A_52_P474528 |  | A_52_P474528 | 1,95 | 1,99 | 1,25 | 0,72 | 2,56 | 1,65 | 1,21 | 0,23 |
| 6 | Irg1 | immunoresponsive gene 1 | A_51_P123625 | 1,94 | 1,87 | 1,57 | 0,47 | 2,88 | 1,72 | 0,65 | 0,60 |
| 6 | Pglyrp2 | peptidoglycan recognition protein 2 | A_51_P102471 | 1,30 | 1,96 | 1,82 | 0,64 | 1,23 | 1,95 | 1,27 | 0,42 |
| 6 | Nppb | natriuretic peptide precursor type B | A_52_P559955 | 1,47 | 1,91 | 1,92 | 0,55 | 1,53 | 1,95 | 1,19 | 0,40 |
| 6 | Il1b | interleukin 1 beta | A_51_P212782 | 1,78 | 1,82 | 1,78 | 0,74 | 2,90 | 1,97 | 1,00 | 0,50 |
| 6 | Ch25h | cholesterol 25-hydroxylase | A_51_P112966 | 1,96 | 1,54 | 1,14 | 0,69 | 2,29 | 1,83 | 0,60 | 0,21 |
| 6 | BC036961 | cDNA sequence BC036961 | A_52_P571746 | 1,54 | 1,62 | 1,83 | 0,87 | 1,72 | 1,39 | 1,09 | 0,51 |
| 6 | Prok2 | prokineticin 2 | A_52_P158476 | 1,16 | 1,85 | 1,96 | 0,26 | 1,09 | 1,36 | 0,70 | 0,03 |
| 6 | Prok2 | prokineticin 2 | A_51_P429252 | 1,14 | 1,84 | 1,97 | 0,23 | 1,18 | 1,38 | 0,68 | 0,16 |
| 6 | BC036961 | cDNA sequence BC036961 | A_51_P340038 | 1,53 | 1,64 | 1,81 | 0,82 | 1,94 | 1,66 | 1,28 | 0,60 |
| 6 | Il8rb | interleukin 8 receptor | A_52_P559975 | 1,43 | 1,80 | 1,68 | 0,90 | 2,12 | 2,09 | 1,31 | 0,81 |
| 6 | A_52_P525348 |  | A_52_P525348 | 0,51 | 1,40 | 2,01 | 0,78 | 0,24 | 0,63 | 0,53 | 0,63 |
| 6 | Ccr7 | chemokine (C-C motif) receptor 7 | A_51_P420229 | 0,81 | 1,59 | 1,96 | 0,64 | 0,31 | 1,29 | 0,90 | 0,16 |
| 6 | Bcl3 | B-cell leukemia/lymphoma 3 | A_51_P136542 | 1,60 | 1,71 | 1,66 | 1,04 | 1,72 | 1,29 | 0,87 | 0,54 |
| 6 | Tnfaip6 | tumor necrosis factor alpha induced protein 6 | A_51_P315785 | 1,90 | 1,44 | 1,36 | 0,51 | 2,68 | 1,43 | 0,42 | 0,54 |
| 6 | E030010A14 | hypothetical protein E030010A14 | A_52_P141608 | 1,56 | 1,41 | 1,79 | 0,92 | 1,02 | 0,59 | 0,68 | 0,39 |
| 6 | Il4ra | interleukin 4 receptor | A_51_P464478 | 1,30 | 1,80 | 1,75 | 0,73 | 1,15 | 1,03 | 0,57 | 0,32 |
| 6 | Tmem8 | transmembrane protein 8 (five membrane-spanning domains) | A_52_P167382 | 0,87 | 1,82 | 1,90 | 0,58 | 0,21 | 0,97 | 0,41 | 0,35 |
| 6 | Socs3 | suppressor of cytokine signaling 3 | A_51_P474459 | 1,66 | 1,77 | 1,68 | 0,57 | 2,36 | 1,45 | 0,94 | 0,24 |
| 6 | Mmp13 | matrix metalloproteinase 13 | A_51_P184484 | 1,11 | 1,97 | 1,50 | 0,15 | 1,55 | 2,11 | 1,91 | -0,16 |
| 6 | Clec4d | C-type lectin domain family 4 | A_51_P383032 | 1,48 | 1,78 | 1,80 | 0,35 | 2,06 | 1,50 | 0,45 | -0,21 |
| 6 | Ccl20 | chemokine (C-C motif) ligand 20 | A_51_P408595 | 1,78 | 1,90 | 0,49 | 0,74 | 2,50 | 2,06 | 0,55 | 0,56 |
| 6 | Plaur | urokinase plasminogen activator receptor | A_52_P681310 | 1,57 | 1,69 | 1,67 | 0,62 | 1,93 | 1,27 | 0,56 | 0,16 |
| 6 | Mmp3 | matrix metalloproteinase 3 | A_51_P255699 | 0,67 | 1,89 | 1,53 | 0,52 | 1,21 | 1,88 | 1,56 | 0,21 |
| 6 | Ccl7 | chemokine (C-C motif) ligand 7 | A_51_P436652 | 1,68 | 1,72 | 1,56 | 0,38 | 2,56 | 1,75 | 0,48 | -0,24 |
| 6 | Adam8 | a disintegrin and metalloprotease domain 8 | A_51_P319917 | 1,39 | 1,66 | 1,58 | 0,81 | 1,90 | 1,69 | 0,91 | 0,42 |
| 6 | Car4 | carbonic anhydrase 4 | A_51_P407028 | 0,74 | 1,74 | 1,77 | 0,64 | 0,87 | 1,70 | 1,15 | 0,78 |
| 6 | Cebpd | CCAAT/enhancer binding protein (C/EBP) | A_51_P444447 | 1,72 | 1,53 | 1,43 | 0,40 | 1,55 | 0,92 | 0,43 | 0,04 |
| 6 | Irg1 | immunoresponsive gene 1 | A_51_P123630 | 1,67 | 1,64 | 1,38 | 0,29 | 2,86 | 1,70 | 0,79 | 0,21 |
| 6 | F10 | coagulation factor X | A_51_P174961 | 1,34 | 1,19 | 1,67 | 0,73 | 1,23 | 0,85 | 0,69 | 0,21 |
| 6 | Chi3l3 | chitinase 3-like 3 | A_51_P167292 | 1,40 | 1,61 | 1,56 | 0,60 | 2,06 | 1,69 | 0,88 | -0,05 |
| 6 | Cyp7b1 | cytochrome P450 | A_51_P461429 | 0,91 | 1,72 | 1,50 | 0,48 | 0,97 | 1,15 | 0,74 | -0,25 |
| 6 | Uox | urate oxidase | A_52_P92037 | 0,64 | 1,78 | 1,56 | 0,34 | 0,43 | 1,35 | 1,07 | -0,01 |
| 6 | Mt1 | metallothionein 1 | A_51_P294979 | 1,52 | 1,56 | 1,43 | 0,62 | 1,63 | 1,04 | 0,58 | 0,23 |
| 6 | Plaur | urokinase plasminogen activator receptor | A_51_P112405 | 1,38 | 1,50 | 1,56 | 0,55 | 1,73 | 1,05 | 0,39 | 0,23 |
| 6 | Stno | strawberry notch homolog (Drosophila) | A_52_P616392 | 1,35 | 1,35 | 1,47 | 0,93 | 1,52 | 0,75 | 0,76 | 0,77 |
| 6 | Ccl12 | chemokine (C-C motif) ligand 12 | A_52_P249514 | 1,20 | 1,45 | 1,57 | 0,46 | 1,40 | 0,75 | -0,12 | 0,14 |
| 6 | Ankrd1 | ankyrin repeat domain 1 (cardiac muscle) | A_52_P120803 | 1,02 | 1,78 | 0,70 | -0,06 | 1,94 | 2,46 | 1,87 | 0,09 |
| 6 | Ccl7 | chemokine (C-C motif) ligand 7 | A_52_P208763 | 1,54 | 1,52 | 1,37 | 0,34 | 2,49 | 1,64 | 0,38 | -0,21 |
| 6 | Cmya1 | cardiomyopathy associated 1 | A_51_P119239 | 1,22 | 1,63 | 1,26 | 0,23 | 2,11 | 1,83 | 1,35 | 0,91 |
| 6 | Ifi202b | interferon activated gene 202B | A_51_P151182 | 1,64 | 1,20 | 0,86 | 0,53 | 2,76 | 1,47 | 0,59 | -0,39 |
| 6 | Kbtbd5 | kelch repeat and BTB (POZ) domain containing 5 | A_51_P235139 | 0,95 | 1,60 | 1,43 | 0,43 | 1,22 | 1,55 | 1,23 | 0,91 |
| 6 | Hbegf | heparin-binding EGF-like growth factor | A_51_P181565 | 1,21 | 1,51 | 1,38 | 0,57 | 1,75 | 1,41 | 0,97 | 0,31 |
| 6 | Serpina9 | serine (or cysteine) proteinase inhibitor | A_51_P131315 | 0,62 | 1,59 | 1,46 | 0,66 | 0,41 | 1,11 | 0,94 | 0,23 |
| 6 | Pyy | peptide YY | A_51_P241733 | 1,62 | 1,49 | 0,11 | 0,96 | 1,55 | 0,92 | 0,51 | 1,25 |
| 6 | Mt1 | metallothionein 1 | A_52_P423810 | 1,57 | 1,24 | 1,28 | 0,23 | 1,94 | 0,69 | 0,03 | -0,08 |
| 6 | Socs1 | suppressor of cytokine signaling 1 | A_51_P279606 | 1,23 | 1,46 | 1,23 | 0,77 | 1,03 | 0,96 | 0,60 | 0,61 |
| 6 | Il4i1 | interleukin 4 induced 1 | A_52_P410449 | 0,88 | 1,39 | 1,52 | 0,64 | 0,74 | 0,96 | 0,72 | 0,49 |
| 6 | Flnc | filamin C | A_51_P205907 | 0,88 | 1,37 | 1,47 | 0,83 | 1,04 | 0,94 | 1,20 | 0,89 |
| 6 | Tmem8 | transmembrane protein 8 (five membrane-spanning domains) | A_52_P196388 | 0,49 | 1,35 | 1,59 | 0,51 | -0,09 | 0,73 | 0,26 | 0,25 |
| 6 | Spp1 | secreted phosphoprotein 1 | A_51_P358765 | 0,47 | 1,72 | 0,83 | -0,10 | 1,03 | 1,35 | 0,49 | -1,11 |
| 6 | Ankrd2 | ankyrin repeat domain 2 (stretch responsive muscle) | A_51_P295315 | 0,46 | 1,12 | 1,64 | 0,35 | 0,53 | 1,27 | 1,34 | 1,08 |
| 6 | Tnc | tenascin C | A_52_P355169 | 0,92 | 1,31 | 1,46 | 0,72 | 0,96 | 1,12 | 0,53 | 0,15 |
| 6 | Uox | urate oxidase | A_51_P205385 | 0,49 | 1,64 | 1,30 | 0,04 | 0,36 | 1,54 | 1,31 | -0,12 |
| 6 | Ltb4r1 | leukotriene B4 receptor 1 | A_51_P109449 | 1,09 | 1,40 | 1,29 | 0,82 | 1,18 | 1,27 | 0,90 | 0,71 |
| 6 | Ccl2 | chemokine (C-C motif) ligand 2 | A_51_P286737 | 1,51 | 1,42 | 1,12 | 0,04 | 2,81 | 1,91 | 0,36 | -0,49 |
| 6 | Fpr1 | formyl peptide receptor 1 | A_51_P312485 | 0,72 | 1,40 | 1,55 | 0,21 | 1,06 | 1,11 | 0,49 | -0,12 |
| 6 | 2010002N04Rik | RIKEN cDNA 2010002N04 gene | A_51_P156857 | 1,07 | 1,33 | 1,39 | 0,78 | 1,15 | 1,02 | 0,53 | 0,28 |
| 6 | Il1f9 | interleukin 1 family | A_51_P408116 | 1,08 | 1,35 | 1,09 | 1,07 | 0,95 | 1,18 | 1,20 | 0,74 |
| 6 | Tnfsf14 | tumor necrosis factor (ligand) superfamily | A_51_P208240 | 1,01 | 1,37 | 1,45 | 0,35 | 1,59 | 1,33 | 0,76 | 0,36 |
| 6 | Rnd1 | Rho family GTPase 1 | A_51_P111164 | 1,44 | 1,32 | 1,21 | 0,24 | 2,22 | 1,13 | 0,68 | 0,44 |
| 6 | Tnfrsf9 | tumor necrosis factor receptor superfamily | A_52_P210511 | 0,86 | 1,42 | 1,46 | 0,39 | 0,78 | 1,38 | 0,78 | 0,13 |
| 6 | Dysfip1 | dysferlin interacting protein 1 | A_51_P189051 | 0,98 | 1,49 | 1,20 | 0,25 | 1,12 | 1,17 | 0,08 | 0,22 |
