# Supplementary figures and images for: Identification of the Rage-dependent gene regulatory network in a mouse model of skin inflammation
Source: BMC Genomics. 2010 Oct 5;11:537. doi: 10.1186/1471-2164-11-537 (PMC3091686; doi:10.1186/1471-2164-11-537)

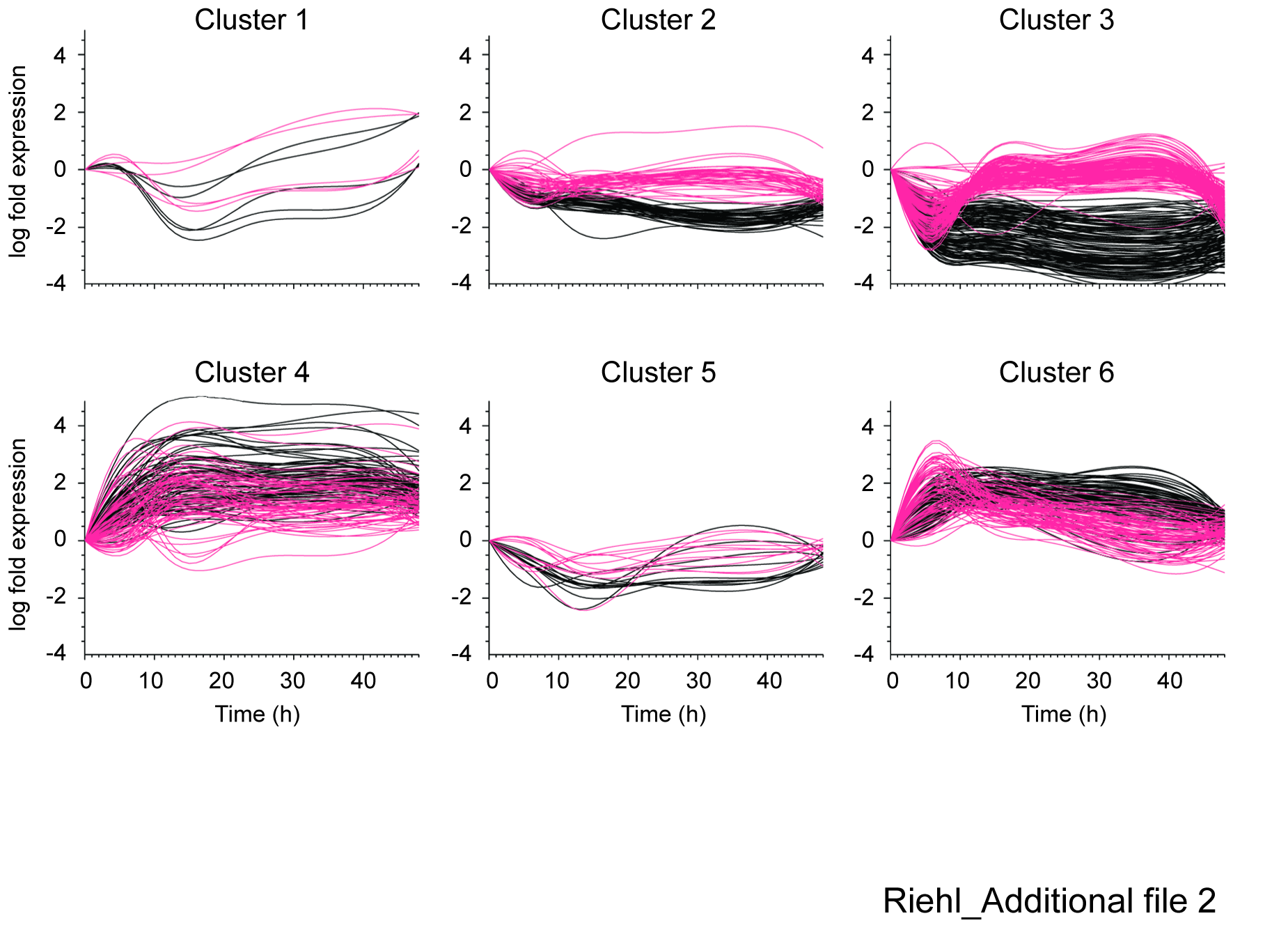

Supplement: Additional file 2 — K-means clustering of TPA-responsive genes. K-means clustering of common TPA-responsive genes in the kinetics of three independent experiments with wt animals revealed 6 clusters. Cluster 1 (n = 5), cluster 2 (n = 45), cluster 3 (n = 125), cluster 4 (n = 71), cluster 5 (n = 11), and cluster 6 (n = 84). Black lines represent transcript levels of genes in wt skin samples. Red lines represent transcript levels in Rage-/- skin samples. [file 1471-2164-11-537-S2.TIFF]

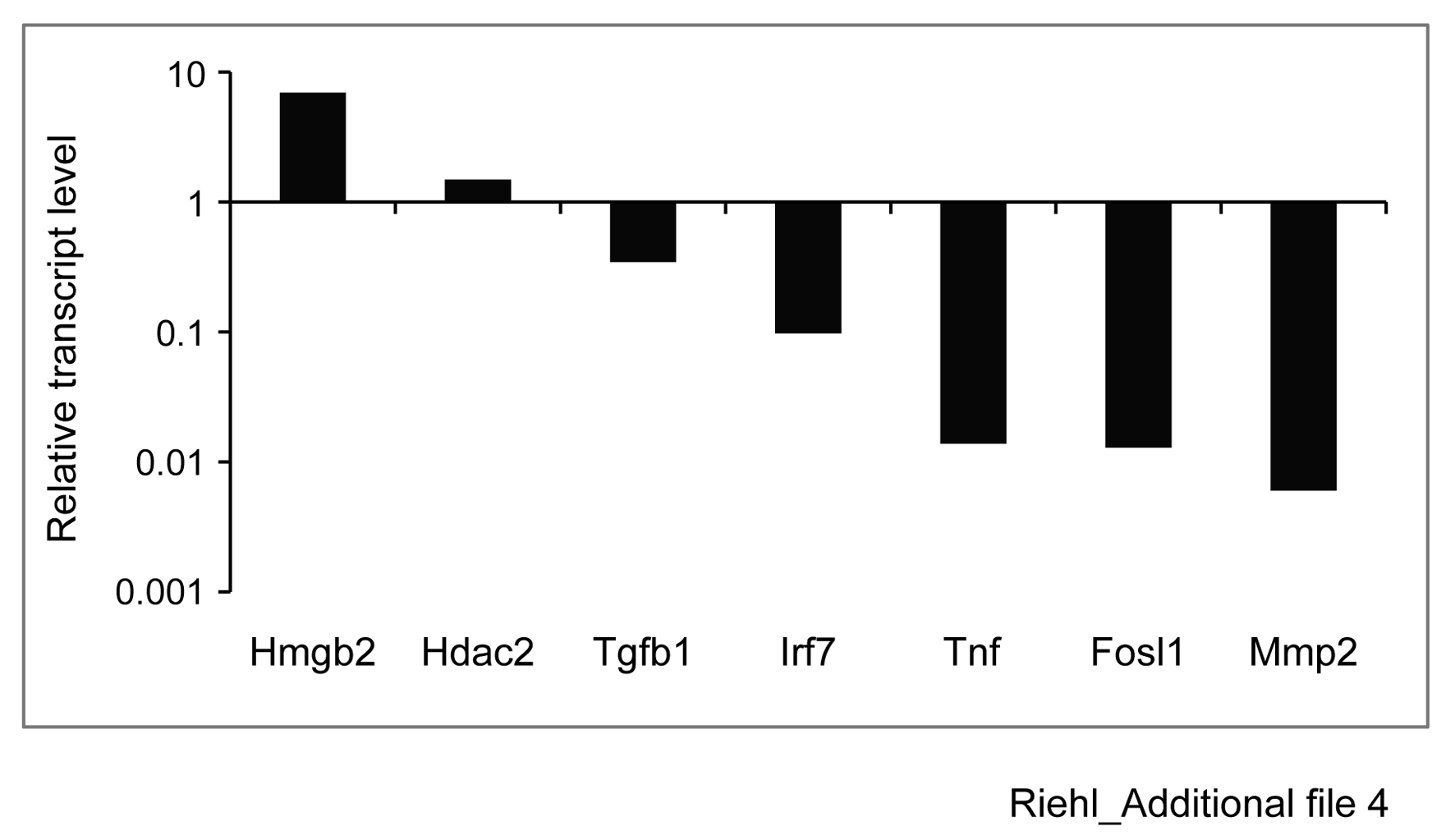

Supplement: Additional file 4 — Quantitative real-time PCR of differentially expressed genes 24 hours after TPA treatment. Relative transcript levels of differentially expressed genes were determined by quantitative real-time PCR with cDNA derived from wt and Rage-/- back skin 24 hours upon TPA treatment. Transcript levels for genes of interest were determined in triplicates with wt and Rage-/- cDNA samples and normalized to Hprt transcript levels. Next, expression values of genes of interest derived from wt cDNA were set to one and bars represent relative transcript levels for Rage-/- cDNA samples. Similar data were obtained for two independent biological replicates (data not shown). [file 1471-2164-11-537-S4.TIFF]

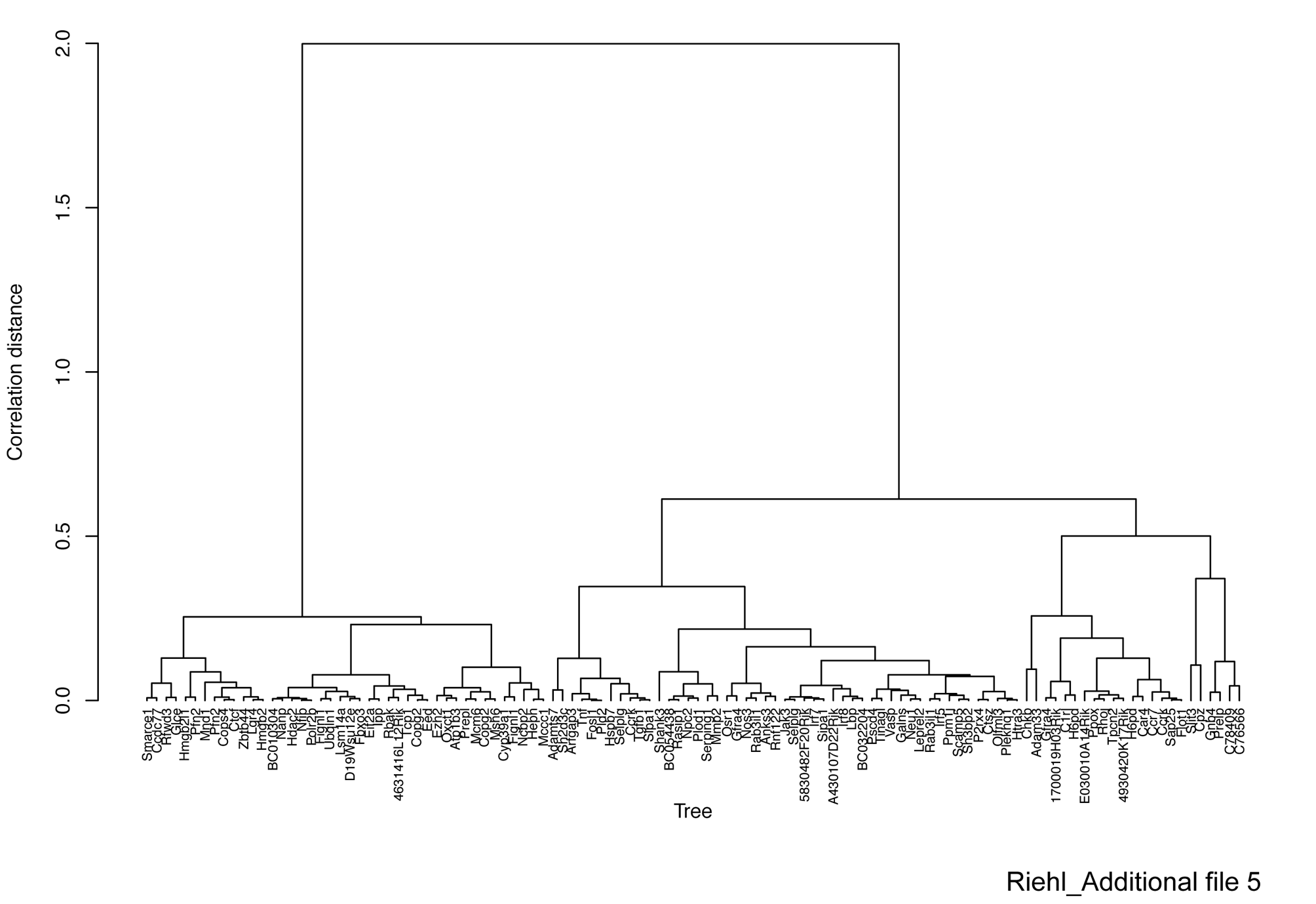

Supplement: Additional file 5 — Cluster dendrogram of genes differentially expressed at t = 24 hours. Clustering was done only over samples from t = 24 hours via Person correlation distance, complete linkage algorithm. Three clusters were defined from the dendrogram. [file 1471-2164-11-537-S5.TIFF]

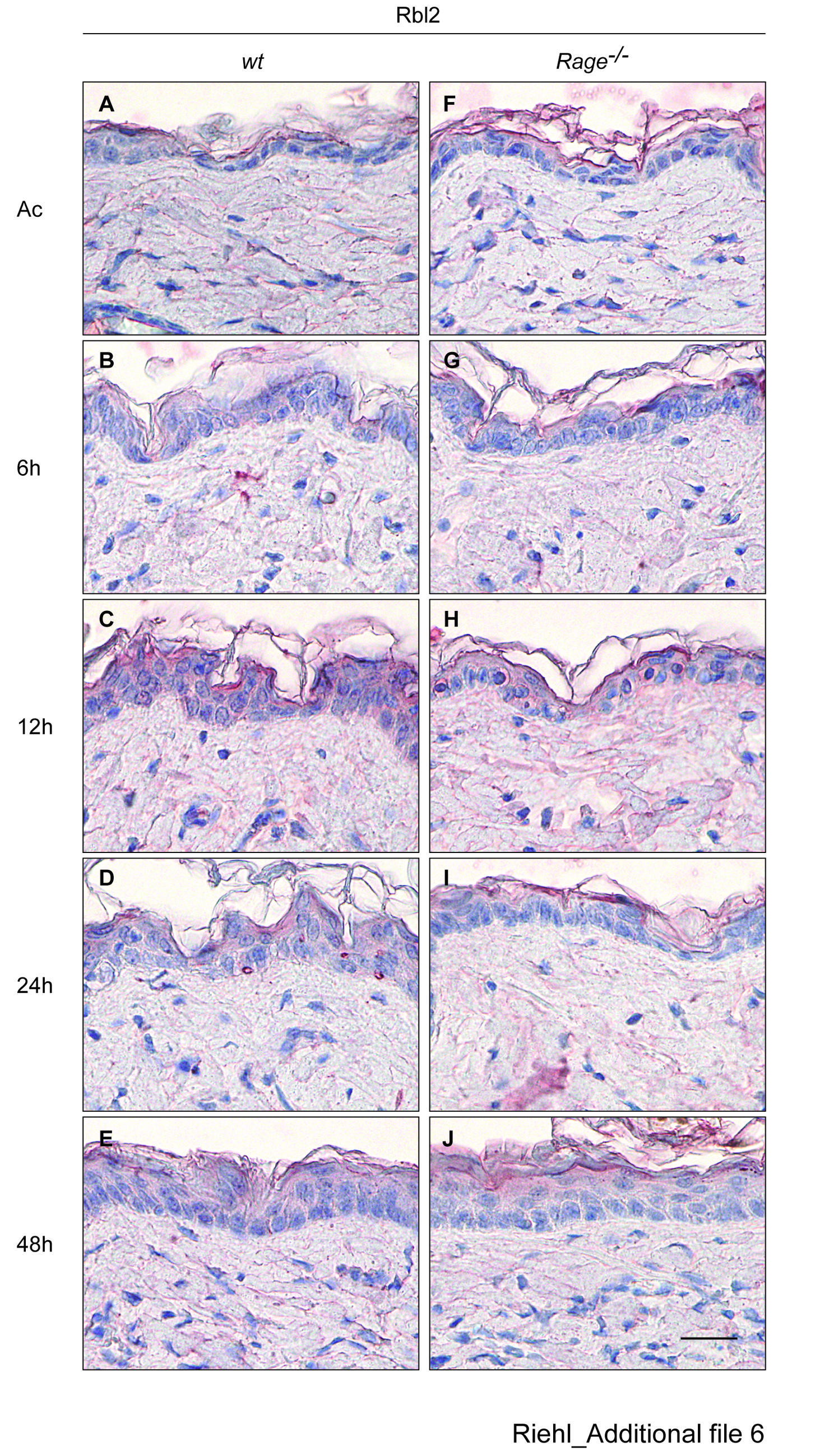

Supplement: Additional file 6 — Rbl2 protein expression in skin following a single TPA stimulus. Tissue sections of acetone- (Ac) or TPA-treated (6, 12, 24, and 48 hours) back skin from wt and Rage-/- mice were analyzed by immunohistochemical staining using Rbl2-specific antibodies. Representative images of at least 2 animals of each genotype and time point are shown with red staining for Rbl2 and counterstaining with hematoxylin. Scale bar = 25 μm. [file 1471-2164-11-537-S6.TIFF]
