# Supplementary material for: Identification of the Rage-dependent gene regulatory network in a mouse model of skin inflammation
Source: BMC Genomics. 2010 Oct 5;11:537. doi: 10.1186/1471-2164-11-537 (PMC3091686; doi:10.1186/1471-2164-11-537)
Supplement: Additional file 3 — Table of 122 genes differentially expressed 24 hours after TPA stimulation. [file 1471-2164-11-537-S3.DOC]

**Additional file 3**: 122 genes differentially expressed 24 hours after TPA stimulation

| **Cluster** | **Symbol** | **Description** | **logFC** | **adj.P.Val** | **Agilent ID** |
| --- | --- | --- | --- | --- | --- |
| 1 | Anks3 | ankyrin repeat and sterile alpha motif domain containing 3 | -0,676 | 0,049 | A_51_P426246 |
| 1 | Npc2 | Niemann Pick type C2 | -0,782 | 0,049 | A_51_P347961 |
| 1 | Rgs19 | regulator of G-protein signaling 19 | -0,808 | 0,049 | A_51_P388984 |
| 1 | Sh3bp2 | SH3-domain binding protein 2 | -0,814 | 0,050 | A_51_P364632 |
| 1 | Vasp | vasodilator-stimulated phosphoprotein | -0,915 | 0,048 | A_51_P179131 |
| 1 | Arfgap3 | ADP-ribosylation factor GTPase activating protein 3 | -0,916 | 0,046 | A_51_P362969 |
| 1 | Ctsz | cathepsin Z | -0,921 | 0,047 | A_52_P62085 |
| 1 | A430107D22Rik | RIKEN cDNA A430107D22 gene | -0,924 | 0,046 | A_52_P16873 |
| 1 | Shank3 | SH3/ankyrin domain gene 3 | -0,932 | 0,047 | A_51_P115626 |
| 1 | 5830482F20Rik | RIKEN cDNA 5830482F20 gene | -0,970 | 0,046 | A_52_P25548 |
| 1 | BC054438 | transmembrane protein 204 | -0,989 | 0,049 | A_52_P288251 |
| 1 | Rnf122 | ring finger protein 122 | -1,011 | 0,047 | A_52_P636742 |
| 1 | Galns | galactosamine (N-acetyl)-6-sulfate sulfatase | -1,019 | 0,049 | A_52_P518160 |
| 1 | Olfml3 | olfactomedin-like 3 | -1,034 | 0,046 | A_51_P191782 |
| 1 | Irf5 | interferon regulatory factor 5 | -1,044 | 0,046 | A_51_P346668 |
| 1 | Tinagl | tubulointerstitial nephritis antigen-like | -1,060 | 0,046 | A_52_P116896 |
| 1 | EG667984 | predicted gene, EG667984 | -1,069 | 0,046 | A_52_P427640 |
| 1 | P2rx4 | purinergic receptor P2X, ligand-gated ion channel 4 | -1,084 | 0,049 | A_51_P308275 |
| 1 | Pld2 | phospholipase D2 | -1,091 | 0,049 | A_51_P357759 |
| 1 | Rasip1 | Ras interacting protein 1 | -1,091 | 0,046 | A_51_P268843 |
| 1 | Nos3 | nitric oxide synthase 3, endothelial cell | -1,104 | 0,046 | A_51_P139651 |
| 1 | Insl3 | Insulin-like 3 | -1,106 | 0,047 | A_51_P288406 |
| 1 | Neu1 | neuraminidase 1 | -1,111 | 0,046 | A_51_P428035 |
| 1 | Jak3 | Janus kinase 3 | -1,111 | 0,049 | A_52_P512847 |
| 1 | Tgfb1 | transforming growth factor, beta 1 | -1,121 | 0,048 | A_51_P390715 |
| 1 | Adamts7 | a disintegrin-like and metallopeptidase (reprolysin type) with thrombospondin type 1 motif, 7 | -1,136 | 0,046 | A_51_P487244 |
| 1 | Osr1 | odd-skipped related 1 (Drosophila) | -1,136 | 0,018 | A_52_P63728 |
| 1 | Lix1l | Lix1-like | -1,148 | 0,046 | A_51_P483351 |
| 1 | Tnf | tumor necrosis factor | -1,150 | 0,046 | A_51_P385099 |
| 1 | Irf8 | interferon regulatory factor 8 | -1,162 | 0,034 | A_51_P187253 |
| 1 | Lrp3 | low density lipoprotein receptor-related protein 3 | -1,163 | 0,049 | A_52_P178866 |
| 1 | Ccrk | cell cycle related kinase | -1,164 | 0,049 | A_51_P433338 |
|  |  | cell cycle related kinase | -1,397 | 0,048 | A_52_P48723 |
| 1 | Adamts2 | a disintegrin-like and metallopeptidase (reprolysin type) with thrombospondin type 1 motif, 2 | -1,178 | 0,046 | A_52_P184042 |
| 1 | Sh2d3c | SH2 domain containing 3C | -1,214 | 0,050 | A_51_P464761 |
| 1 | Scamp5 | secretory carrier membrane protein 5 | -1,239 | 0,048 | A_52_P409731 |
| 1 | Leprel2 | leprecan-like 2 | -1,241 | 0,049 | A_51_P149699 |
| 1 | Sipa1 | signal-induced proliferation associated gene 1 | -1,245 | 0,048 | A_51_P191331 |
|  |  | signal-induced proliferation associated gene 1 | -1,355 | 0,049 | A_52_P675381 |
| 1 | Plekhq1 | pleckstrin homology domain containing, family O member 2 | -1,284 | 0,049 | A_51_P293982 |
| 1 | Plod1 | procollagen-lysine, 2-oxoglutarate 5-dioxygenase 1 | -1,313 | 0,047 | A_51_P320304 |
| 1 | Htra3 | HtrA serine peptidase 3 | -1,327 | 0,046 | A_52_P217437 |
| 1 | Ppm1j | protein phosphatase 1J | -1,334 | 0,046 | A_51_P399175 |
| 1 | Serping1 | serine (or cysteine) peptidase inhibitor, clade G, member 1 | -1,372 | 0,046 | A_51_P376238 |
| 1 | BC032204 | fermitin family homolog 3 (Drosophila) | -1,389 | 0,046 | A_52_P38639 |
| 1 | Hspb7 | heat shock protein family, member 7 (cardiovascular) | -1,396 | 0,049 | A_51_P346445 |
| 1 | Fosl1 | fos-like antigen 1 | -1,404 | 0,049 | A_51_P308796 |
| 1 | Pscd4 | pleckstrin homology, Sec7 and coiled/coil domains 4 | -1,448 | 0,050 | A_51_P231398 |
| 1 | Mmp2 | matrix metallopeptidase 2 | -1,462 | 0,046 | A_51_P341736 |
| 1 | Rab3il1 | RAB3A interacting protein (rabin3)-like 1 | -1,478 | 0,046 | A_52_P450918 |
|  |  | RAB3A interacting protein (rabin3)-like 1 | -1,586 | 0,046 | A_51_P451758 |
| 1 | Gfra4 | glial cell line derived neurotrophic factor family receptor alpha 4 | -1,363 | 0,047 | A_52_P171197 |
|  |  | glial cell line derived neurotrophic factor family receptor alpha 4 | -1,522 | 0,046 | A_51_P241426 |
| 1 | Lbp | lipopolysaccharide binding protein | -1,538 | 0,018 | A_51_P454008 |
| 1 | Selplg | selectin, platelet (p-selectin) ligand | -1,560 | 0,046 | A_51_P131364 |
|  |  | selectin, platelet (p-selectin) ligand | -1,585 | 0,049 | A_51_P131358 |
| 1 | Irf7 | interferon regulatory factor 7 | -1,624 | 0,046 | A_51_P421876 |
| 2 | Pcdhga12 | protocadherin gamma subfamily A, 9 | -0,756 | 0,046 | A_51_P470769 |
| 2 | Slit3 | slit homolog 3 (Drosophila) | -0,840 | 0,049 | A_51_P301809 |
| 2 | 4930420K17Rik | RIKEN cDNA 4930420K17 gene | -0,847 | 0,046 | A_52_P668984 |
| 2 | Tpcn2 | two pore segment channel 2 | -0,865 | 0,049 | A_52_P135155 |
| 2 | 1810006K21Rik | RIKEN cDNA 1810006K21 gene | -0,895 | 0,049 | A_52_P17635 |
| 2 | 1700019H03Rik | RIKEN cDNA 1700019H03 gene | -0,903 | 0,049 | A_51_P116298 |
| 2 | H6pd | hexose-6-phosphate dehydrogenase (glucose 1-dehydrogenase) | -0,917 | 0,048 | A_51_P321643 |
|  |  | hexose-6-phosphate dehydrogenase (glucose 1-dehydrogenase) | -1,328 | 0,048 | A_52_P83549 |
| 2 | Lgals4 | lectin, galactose binding, soluble 4 | -0,934 | 0,049 | A_52_P634250 |
| 2 | C78409 | expressed sequence C78409 | -0,954 | 0,049 | A_52_P140881 |
| 2 | Gnb4 | guanine nucleotide binding protein (G protein), beta 4 | -0,957 | 0,046 | A_51_P464308 |
| 2 | Ppox | protoporphyrinogen oxidase | -0,970 | 0,046 | A_51_P310949 |
| 2 | Rhoj | ras homolog gene family, member J | -0,974 | 0,046 | A_52_P592466 |
| 2 | Prelp | proline arginine-rich end leucine-rich repeat | -0,988 | 0,047 | A_52_P486260 |
| 2 | C76566 | expressed sequence C76566 | -1,028 | 0,049 | A_51_P389864 |
| 2 | C1rl | complement component 1, r subcomponent-like | -1,074 | 0,049 | A_51_P251367 |
| 2 | Lrch4 | leucine-rich repeats and calponin homology (CH) domain containing 4 | -1,116 | 0,046 | A_52_P38964 |
| 2 | Flot1 | flotillin 1 | -1,126 | 0,046 | A_51_P423091 |
| 2 | Chkb | choline kinase beta | -1,151 | 0,046 | A_52_P35304 |
| 2 | Car4 | carbonic anhydrase 4 | -1,223 | 0,019 | A_51_P407028 |
| 2 | Adam33 | a disintegrin and metallopeptidase domain 33 | -1,302 | 0,046 | A_52_P333289 |
| 2 | Cpz | carboxypeptidase Z | -1,323 | 0,047 | A_51_P255675 |
| 2 | E030010A14Rik | RIKEN cDNA E030010A14 gene | -1,410 | 0,022 | A_52_P141608 |
| 2 | H2-Ab1 | histocompatibility 2, class II antigen A, beta 1 | -1,468 | 0,049 | A_51_P215242 |
| 2 | Ccr7 | chemokine (C-C motif) receptor 7 | -1,486 | 0,048 | A_51_P420229 |
| 2 | Gtl2 | GTL2, imprinted maternally expressed untranslated mRNA | -1,655 | 0,049 | A_52_P391505 |
| 3 | Eif2a | eukaryotic translation initiation factor 2a | 1,736 | 0,046 | A_52_P620793 |
| 3 | 1190002N15Rik | RIKEN cDNA 1190002N15 gene | 1,675 | 0,048 | A_52_P394291 |
| 3 | Oxct1 | 3-oxoacid CoA transferase 1 | 1,574 | 0,046 | A_51_P107326 |
| 3 | Fignl1 | fidgetin-like 1 | 1,526 | 0,046 | A_51_P383489 |
|  |  | fidgetin-like 1 | 1,339 | 0,049 | A_52_P148553 |
| 3 | Lgr4 | leucine-rich repeat-containing G protein-coupled receptor 4 | 1,517 | 0,047 | A_52_P276727 |
| 3 | Nanp | N-acetylneuraminic acid phosphatase | 1,366 | 0,046 | A_52_P354432 |
| 3 | Nfib | nuclear factor I/B | 1,275 | 0,046 | A_51_P304412 |
| 3 | Fbxo3 | F-box protein 3 | 1,270 | 0,046 | A_52_P113350 |
| 3 | Pfn2 | profilin 2 | 1,245 | 0,049 | A_51_P391466 |
|  |  | profilin 2 | 1,100 | 0,046 | A_52_P359061 |
| 3 | Atp1b3 | ATPase, Na+/K+ transporting, beta 3 polypeptide | 1,244 | 0,049 | A_51_P520378 |
| 3 | Mcm6 | minichromosome maintenance deficient 6 (MIS5 homolog, S. pombe) (S. cerevisiae) | 1,229 | 0,046 | A_51_P360492 |
| 3 | Cops4 | COP9 (constitutive photomorphogenic) homolog, subunit 4 (Arabidopsis thaliana) | 1,178 | 0,048 | A_51_P371119 |
| 3 | Eed | embryonic ectoderm development | 1,171 | 0,049 | A_51_P238643 |
| 3 | Hdac2 | histone deacetylase 2 | 1,167 | 0,049 | A_51_P116007 |
| 3 | Ermp1 | endoplasmic reticulum metallopeptidase 1 | 1,147 | 0,046 | A_52_P618745 |
| 3 | Polr2b | polymerase (RNA) II (DNA directed) polypeptide B | 1,144 | 0,046 | A_51_P509881 |
| 3 | Mnd1 | meiotic nuclear divisions 1 homolog (S. cerevisiae) | 1,133 | 0,048 | A_51_P155966 |
| 3 | Heph | hephaestin | 1,133 | 0,046 | A_51_P466270 |
| 3 | Strn | striatin, calmodulin binding protein | 1,081 | 0,046 | A_51_P195982 |
| 3 | Glce | glucuronyl C5-epimerase | 1,045 | 0,046 | A_51_P337543 |
| 3 | Cyp39a1 | cytochrome P450, family 39, subfamily a, polypeptide 1 | 1,045 | 0,049 | A_51_P515446 |
| 3 | Aph1b | anterior pharynx defective 1b homolog (C. elegans) | 1,043 | 0,048 | A_51_P325124 |
| 3 | Ccny | cyclin Y | 1,035 | 0,047 | A_51_P358776 |
| 3 | Msh6 | mutS homolog 6 (E. coli) | 1,028 | 0,046 | A_51_P405565 |
| 3 | Anp32e | acidic (leucine-rich) nuclear phosphoprotein 32 family, member E | 1,010 | 0,046 | A_52_P125350 |
| 3 | Hmgb2 | high mobility group box 2 | 1,009 | 0,046 | A_52_P485417 |
| 3 | BC010304 | cDNA sequence BC010304 | 0,986 | 0,046 | A_51_P387845 |
| 3 | Mccc1 | methylcrotonoyl-Coenzyme A carboxylase 1 (alpha) | 0,961 | 0,046 | A_51_P282975 |
| 3 | Zbtb44 | zinc finger and BTB domain containing 44 | 0,936 | 0,049 | A_51_P303000 |
| 3 | Copg2 | coatomer protein complex, subunit gamma 2 | 0,927 | 0,046 | A_52_P48777 |
|  |  | coatomer protein complex, subunit gamma 2 | 0,859 | 0,046 | A_51_P172692 |
| 3 | Ipp | IAP promoted placental gene | 0,926 | 0,046 | A_51_P340816 |
| 3 | Map4k3 | mitogen-activated protein kinase kinase kinase kinase 3 | 0,903 | 0,046 | A_51_P504602 |
| 3 | Ncbp2 | nuclear cap binding protein subunit 2 | 0,874 | 0,047 | A_51_P151433 |
| 3 | Smarce1 | SWI/SNF related, matrix associated, actin dependent regulator of chromatin, subfamily e, member 1 | 0,857 | 0,046 | A_52_P182741 |
| 3 | Ctcf | CCCTC-binding factor | 0,851 | 0,046 | A_51_P513785 |
| 3 | Ubqln1 | ubiquilin 1 | 0,830 | 0,046 | A_52_P322658 |
| 3 | Lsm14a | LSM14 homolog A (SCD6, S. cerevisiae) | 0,828 | 0,046 | A_51_P320860 |
| 3 | Rfwd3 | ring finger and WD repeat domain 3 | 0,822 | 0,046 | A_52_P4698 |
| 3 | Ezh2 | enhancer of zeste homolog 2 (Drosophila) | 0,808 | 0,046 | A_51_P248067 |
| 3 | Hmgb2l1 | high mobility group box 2-like 1 | 0,760 | 0,048 | A_51_P323174 |
| 3 | 4631416L12Rik | RIKEN cDNA 4631416L12 gene | 0,749 | 0,049 | A_51_P473953 |
| 3 | Rbak | RB-associated KRAB repressor | 0,729 | 0,046 | A_52_P192307 |
| 3 | Ccdc77 | coiled-coil domain containing 77 | 0,706 | 0,048 | A_51_P178545 |
| 3 | Prepl | prolyl endopeptidase-like | 0,627 | 0,047 | A_52_P612079 |
| 3 | Sap18 | Sin3-associated polypeptide 18 | 0,610 | 0,046 | A_51_P386031 |
