# Supplementary material for: Identification of the Rage-dependent gene regulatory network in a mouse model of skin inflammation
Source: BMC Genomics. 2010 Oct 5;11:537. doi: 10.1186/1471-2164-11-537 (PMC3091686; doi:10.1186/1471-2164-11-537)
Supplement: Additional file 7 — Table of primer sequences used for quantitative real-time PCR analysis. [file 1471-2164-11-537-S7.DOC]

**Additional file 7.** Primer pairs used for RQ-PCR analysis

| **Name** | **Forward primer 5´- 3´** | **Reverse primer 5´- 3´** |
| --- | --- | --- |
| *Fosl1* | TTGCTCCTCCGCTCACCGAAAGA | AGGCCTGCTTGGATCAATGGGAGAA |
| *Hdac2* | TGCTGCAGTGTGGCGCAGAC | TCCACCGAGCATCAGCAATGGC |
| *Hmgb2* | CCGCGACTGGTCTGGTCAAGTTGC | ACGGCGCGGCGTCTGTCTAC |
| *Irf7* | GCTGCTGAGCGAAGAGAGCG | ACACACCCTGTGTGGGCAGA |
| *Mmp2* | CCACGATGGCAAGGTGTGGT | AATGCCAAGTTCTTGGTGTAGGTG |
| *Tgfb1* | GGGCGTCTCAAGAAGCAAAAG | AGGGGGCGTACACAGCAGTT |
| *Tnf* | GCCTCTTCTCATTCCTGCTT | CTCCTCCACTTGGTGGTTTG |
