# Supplementary material for: Identification of the Rage-dependent gene regulatory network in a mouse model of skin inflammation
Source: BMC Genomics. 2010 Oct 5;11:537. doi: 10.1186/1471-2164-11-537 (PMC3091686; doi:10.1186/1471-2164-11-537)
Supplement: Additional file 8 — Table of primary antibodies used for Western Blot (WB) and immunohistochemistry analysis (IHC). [file 1471-2164-11-537-S8.DOC]

**Additional file 8: Primary antibodies used for Western Blot (WB) and immunohistochemistry analysis (IHC)**

**Antibody Dilution Commerical Source**

rabbit anti-E2f1 (C-20), sc193 1:50 (IHC); 1:1000 (WB) Santa Cruz Biotechnology, Santa Cruz, CA

rabbit anti-E2f4 (A-20), sc1082 1:50 (IHC); 1:1000 (WB) Santa Cruz Biotechnology, Santa Cruz, Ca

rabbit anti-Rb, #53823 1:75 (IHC); 1:150 (WB) Anaspec, San Jose, CA

rabbit anti-p107 (C-18), sc318 (Rbl1) 1: 50 (IHC); 1:500 (WB) Santa Cruz Biotechnology, Santa Cruz, CA

rabbit anti-p130 (C-20), sc317 (Rbl2) 1:50 (IHC) Santa Cruz Biotechnology, Santa Cruz, CA

goat anti-actin (C-11), sc1615 1:1000 (WB) Santa Cruz Biotechnology, Santa Cruz, CA
